# IVIVE: Facilitating the Use of *In Vitro* Toxicity Data in Risk Assessment and Decision Making

**DOI:** 10.3390/toxics10050232

**Published:** 2022-05-01

**Authors:** Xiaoqing Chang, Yu-Mei Tan, David G. Allen, Shannon Bell, Paul C. Brown, Lauren Browning, Patricia Ceger, Jeffery Gearhart, Pertti J. Hakkinen, Shruti V. Kabadi, Nicole C. Kleinstreuer, Annie Lumen, Joanna Matheson, Alicia Paini, Heather A. Pangburn, Elijah J. Petersen, Emily N. Reinke, Alexandre J. S. Ribeiro, Nisha Sipes, Lisa M. Sweeney, John F. Wambaugh, Ronald Wange, Barbara A. Wetmore, Moiz Mumtaz

**Affiliations:** 1 Inotiv-RTP, 601 Keystone Park Drive, Suite 200, Morrisville, NC 27560, USA; xiaoqing.chang@inotivco.com (X.C.); dave.allen@inotivco.com (D.G.A.); shannon.bell@inotivco.com (S.B.); lauren.browning@icf.com (L.B.); patricia.ceger@inotivco.com (P.C.); 2 U.S. Environmental Protection Agency, Office of Pesticide Programs, 109 T.W. Alexander Drive, Durham, NC 27709, USA; tan.cecilia@epa.gov; 3 U.S. Food and Drug Administration, Center for Drug Evaluation and Research, 10903 New Hampshire Avenue, Silver Spring, MD 20903, USA; paul.brown@fda.hhs.gov (P.C.B.); axribeiro@hovione.com (A.J.S.R.); ronald.wange@fda.hhs.gov (R.W.); 4 The Henry M. Jackson Foundation, Air Force Research Laboratory, 711 Human Performance Wing, Wright-Patterson Air Force Base, OH 45433, USA; jgearhart@hjfresearch.org; 5 National Library of Medicine, National Center for Biotechnology Information, 8600 Rockville Pike, Bethesda, MD 20894, USA; pertti.hakkinen@nih.gov; 6 U.S. Food and Drug Administration, Center for Food Safety and Applied Nutrition, Office of Food Additive Safety, 5001 Campus Drive, HFS-275, College Park, MD 20740, USA; shruti.kabadi@fda.hhs.gov; 7 National Institute of Environmental Health Sciences, National Toxicology Program Interagency Center for the Evaluation of Alternative Toxicological Methods, P.O. Box 12233, Research Triangle Park, NC 27709, USA; nicole.kleinstreuer@nih.gov; 8 U.S. Food and Drug Administration, National Center for Toxicological Research, 3900 NCTR Road, Jefferson, AR 72079, USA; alumen@amgen.com; 9 U.S. Consumer Product Safety Commission, Division of Toxicology and Risk Assessment, 5 Research Place, Rockville, MD 20850, USA; jmatheson@cpsc.gov; 10 European Commission, Joint Research Centre (JRC), 21027 Ispra, Italy; alicia.paini@esqlabs.com; 11 Air Force Research Laboratory, 711 Human Performance Wing, 2729 R Street, Area B, Building 837, Wright-Patterson Air Force Base, OH 45433, USA; heather.pangburn.1@us.af.mil; 12 U.S. Department of Commerce, National Institute of Standards and Technology, 100 Bureau Drive, Gaithersburg, MD 20899, USA; elijah.petersen@nist.gov; 13 U.S. Army Public Health Center, 8252 Blackhawk Rd., Aberdeen Proving Ground, MD 21010, USA; emily.n.reinke.civ@mail.mil; 14 U.S. Environmental Protection Agency, Center for Computational Toxicology and Exposure, 109 TW Alexander Dr., Research Triangle Park, NC 27711, USA; sipes.nisha@epa.gov (N.S.); wambaugh.john@epa.gov (J.F.W.); wetmore.barbara@epa.gov (B.A.W.); 15 UES, Inc., 4401 Dayton-Xenia Road, Beavercreek, OH 45432, Assigned to Air Force Research Laboratory, 711 Human Performance Wing, Wright-Patterson Air Force Base, OH 45433, USA; lisa.sweeney.3.ctr@us.af.mil; 16 Agency for Toxic Substances and Disease Registry, Office of the Associate Director for Science, 1600 Clifton Road, S102-2, Atlanta, GA 30333, USA

**Keywords:** absorption, distribution, metabolism, excretion (ADME), dosimetry, *in vitro* to *in vivo* extrapolation (IVIVE), physiologically based pharmacokinetic (PBPK) model, Interagency Coordinating Committee on the Validation of Alternative Methods (ICCVAM), new approach methodologies (NAMs), risk assessment, toxicity tests

## Abstract

During the past few decades, the science of toxicology has been undergoing a transformation from observational to predictive science. New approach methodologies (NAMs), including *in vitro* assays, *in silico* models, read-across, and *in vitro* to *in vivo* extrapolation (IVIVE), are being developed to reduce, refine, or replace whole animal testing, encouraging the judicious use of time and resources. Some of these methods have advanced past the exploratory research stage and are beginning to gain acceptance for the risk assessment of chemicals. A review of the recent literature reveals a burst of IVIVE publications over the past decade. In this review, we propose operational definitions for IVIVE, present literature examples for several common toxicity endpoints, and highlight their implications in decision-making processes across various federal agencies, as well as international organizations, including those in the European Union (EU). The current challenges and future needs are also summarized for IVIVE. In addition to refining and reducing the number of animals in traditional toxicity testing protocols and being used for prioritizing chemical testing, the goal to use IVIVE to facilitate the replacement of animal models can be achieved through their continued evolution and development, including a strategic plan to qualify IVIVE methods for regulatory acceptance.

## 1. Introduction

U.S. regulatory and public health agencies are charged with protecting human, animal, and environmental health. Agencies evaluate potential risks presented by substances that enter the environment, such as chemicals, engineered nanomaterials, industrial chemicals, metals and metalloids, pharmaceuticals, microplastics, or their degradation products or metabolites. Risk evaluations often involve the use of toxicological tests conducted in living organisms. However, concerns have been raised about these tests, including animal welfare, the time and cost they require, and the ability of using animal models to accurately represent human effects. These concerns have led to an increasing interest in developing alternative methods that are rapid and efficient and that replace, reduce, or refine (3Rs) animal use [1]. Efforts to achieve these goals have resulted in the 2016 amendment to the Toxic Substances Control Act that encouraged the development of new approach methodologies (NAMs) to inform chemical hazard and risk assessment [2,3]. NAMs can include *in silico*, *in chemico*, and *in vitro* approaches [4], and the application of NAMs is increasing, as federal agencies and international entities have started adopting them, in some contexts, to reduce or phase out animal testing. For example, the U.S. Environmental Protection Agency (EPA) is the first U.S. agency to announce plans to redirect funds towards the development of NAMs and away from animal testing. *In vitro* to *in vivo* extrapolation (IVIVE) can be considered a NAM because it is broadly defined as a quantitative or qualitative transposition of *in vitro* experimental data to predict *in vivo* phenomena [5,6,7].

### 1.1. Multiple Definitions of IVIVE in Literature

In the literature, the term “IVIVE” can be found to generally refer to two different processes. Traditionally, the term IVIVE is used to refer to estimating *in vivo* whole-organ absorption, distribution, metabolism, and excretion (ADME) properties by scaling from properties measured *in vitro*, which is often used when constructing a bottom-up pharmacokinetic (PK) or physiologically based (pharmaco-) kinetic (PB(P)K) model [8,9,10,11,12]. The ADME parameters most commonly measured *in vitro* are the hepatic metabolism, plasma protein binding fraction, and intestinal absorption [13,14,15,16,17,18]. *In vitro* methods are also available for measuring other parameters, such as p-glycoprotein-mediated efflux ratio [19,20], renal clearance [21,22], extrahepatic clearance [23], glucuronidation [24,25], and tissue or blood partition coefficients [26].

Recently, the term IVIVE has been used to describe the process of converting an *in vitro* concentration associated with bioactivity to an external exposure level [27,28]. This process is also referred to as reverse dosimetry or reverse toxicokinetics, which involves using a PK model to determine a plausible exposure level that leads to a tissue or plasma concentration equivalent to the *in vitro* concentration [29,30]. The predicted exposure level can then be compared with the actual or estimated human exposures to estimate potential health risks [31]. To distinguish this definition from the first definition, some used the term quantitative IVIVE (QIVIVE) [32]. However, some usages of the term IVIVE are broadened to cover both meanings. Therefore, it is suggested to refer to the context in which the IVIVE is applied when using the term IVIVE. In this review, to avoid confusion, we use “IVIVE of ADME parameters” to refer specifically to the traditional interpretation, and “IVIVE of dosimetry” to refer specifically to the second and recent definition. When both processes are involved and it is not easy to distinguish one term from another, we will use the term IVIVE with a detailed context. 

IVIVE of dosimetry typically assumes that chemicals in an *in vitro* system behave the same way they behave in blood or tissue in an organism. However, this assumption may not be appropriate due to several *in vitro* kinetic factors, such as chemical binding to proteins and lipids in the cell culture medium, evaporation, binding to plastic containers, uptake into the cultured cells, and degradation processes [33,34,35,36,37]. An *in vitro* bioactivity concentration may be adjusted for these kinetic factors or assumed equivalent to an *in vivo* plasma or tissue concentration. Then, pharmacokinetic models, such as PBPK models [24], are used to convert the plasma or tissue concentration to an external dose. These models include parameters that describe the ADME processes, and the values of model parameters may be obtained using *in vitro* assays [38,39,40] and *in silico* methods, such as quantitative structure–activity relationship (QSAR) models [41].

In some instances, when combining PK and pharmacodynamic (PD) modeling, IVIVE of dosimetry can be used to predict *in vivo* organ toxicity levels based on *in vitro* toxicity testing results or translate the *in vitro* concentration–response curve to an external dose–response curve [6,39,42,43,44]. In these cases, the term IVIVE more broadly refers to extrapolating an *in vitro* bioactivity measurement that represents a molecular initiating event or a battery of *in vitro* assays that interrogate multiple elements in a toxicity pathway to an *in vivo* toxicological endpoint. Such a practice is likely to remain an important challenge in conducting safety assessments based on *in vitro* toxicity testing [45,46].

### 1.2. Overview of Regulatory Applications of IVIVE

Several regulatory agencies have considered applying IVIVE of dosimetry to use *in vitro* bioactivity data in assessing human health risks from chemical exposure. For example, the EPA utilized data from *in vitro* high-throughput screening (HTS) assays in Toxicology in the 21st Century (Tox21) [47] and Toxicity Forecaster (ToxCast^TM^) [48] and IVIVE to prioritize chemicals for further testing under the Endocrine Disruptor Screening Program [49]. The Organisation for Economic Co-operation and Development (OECD) Guidance Document on Good In Vitro Method Practices (GIVIMP) describes the process of conducting IVIVE to enable animal-free risk assessment [50]. The workflow known as “Next Generation Risk Assessment” illustrates a process for chemical safety assessment that is determined entirely by *in vitro* testing and IVIVE [51,52]. IVIVE was also included in a recent OECD case study on the use of integrated approaches to testing and assessment (IATA) of developmental neurotoxicity modalities [53]. 

There are also several guidance documents related to IVIVE of ADME parameters. For example, OECD published [54] a guidance on the determination of intrinsic clearance using cryopreserved hepatocytes or liver S9 sub-cellular fractions from rainbow trout. A European Union Reference Laboratory for Alternatives to Animal Testing (EURL ECVAM) 2016 workshop to facilitate the acceptance and use of new generation PBK models in the regulatory domain [55,56] highlighted the need to develop guidance on constructing PBK models using *in vitro* and *in silico* data. Moreover, the U.S. Food and Drug Administration (FDA) recommends advancing PBPK modeling and IVIVE to address various shortcomings that limit the utility of NAMs, such as microphysiological systems models, as a replacement for whole animal toxicity testing of human pharmaceuticals for regulatory purposes or as an improvement in the predictivity of the testing [57].

### 1.3. Introduction to the IVIVE Workgroup

Given the critical role of IVIVE in using NAMs to supplement or replace the current toxicity testing methods, an IVIVE workgroup (IVIVE–WG) was established under the Interagency Coordinating Committee on the Validation of Alternative Methods (ICCVAM) to help actualize and implement the ICCVAM Strategic Roadmap, which requires federal agencies and stakeholders to work together to develop and implement NAMs to toxicity testing that improve human health relevance and reduce or eliminate the need for testing in animals [3]. The IVIVE–WG includes representatives from nine U.S. federal offices: the Agency for Toxic Substances and Disease Registry (ATSDR), the Consumer Product Safety Commission (CPSC), the Department of Defense (DoD), the National Library of Medicine (NLM), the Environmental Protection Agency (EPA), the Food and Drug Administration (FDA), the National Institute of Environmental Health Sciences (NIEHS), the National Institute of Standards and Technology (NIST), and the Department of Labor’s Occupational Safety and Health Administration (OSHA). International partners participating in the IVIVE workgroup include EURL ECVAM (which is part of the European Commission’s Joint Research Centre) and the Japanese Center for the Validation of Alternative Methods (JaCVAM). The workgroup was charged with cataloging and evaluating currently available IVIVE approaches, and its activities have focused on harmonizing the technical terms used in IVIVE applications, evaluating the suitability of IVIVE approaches for specific research or regulatory purposes, and assessing whether additional tools or models are needed.

This manuscript presents the workgroup’s findings on the judicious use of IVIVE and its potential to support decision making. We review the various applications of IVIVE found in published peer-reviewed literature and highlight examples to demonstrate the use of IVIVE in the safety assessment of drugs, food substances, and environmental chemicals. We have also compiled a non-exhaustive list of resources and tools to support IVIVE, and present areas of research needs and future opportunities.

## 2. Methods

Members of the IVIVE–WG provided input on their respective agencies’ specific risk assessment applications that can involve the IVIVE approach; agency-specific guidance documents or publications that are related to IVIVE; modeling tools or software an agency plans to use or has used for facilitating IVIVE analysis and decision making; as well as agency needs, data gaps, or uncertainty that prevents using IVIVE in regulatory risk assessment. Input was received from the ATSDR, CPSC, DoD (U.S. Army Public Health Center and U.S. Air Force, 711 Human Performance Wing, Airman Biosciences Division (RHB)), NLM, EPA, FDA, NIEHS, and EURL ECVAM, and the responses are summarized in following tables.

In addition to ICCVAM member agencies’ inputs, a literature review was conducted to help grasp the extent IVIVE is used in the broader scientific community. The terms “*In vitro* to *in vivo* extrapolation”, or “IVIVE”, were used to search for literature in PubMed, Scopus, and Web of Science in May 2020. In addition, to harmonize IVIVE-related vocabularies in the literature and to ensure better communication of IVIVE concepts in a precise and consistent manner, a glossary of controlled vocabulary for IVIVE was developed by the IVIVE–WG (Table S1).

## 3. Regulatory Application of IVIVE

Input on agency-specific IVIVE activities is summarized in the tables below. Table 1 lists each agency’s specific risk assessment applications that can involve the use of IVIVE. The ATSDR, NIEHS/NTP, NIST, DoD (except in limited internal capacities) and EURL ECVAM are not regulatory agencies, so they only use IVIVE for non-regulatory applications if it is applied. Table 2 summarizes the guidance documents and publications that describe applications of IVIVE. In Table 2, in addition to inputs from the IVIVE–WG members, we also added the recently published science approach document from Health Canada on the use of an *in vitro*-based point of departure (POD) as a conservative surrogate in the absence of traditional hazard data [58].

## 4. Applications of IVIVE Approaches

### 4.1. Review of IVIVE Literature

**Figure 1:**
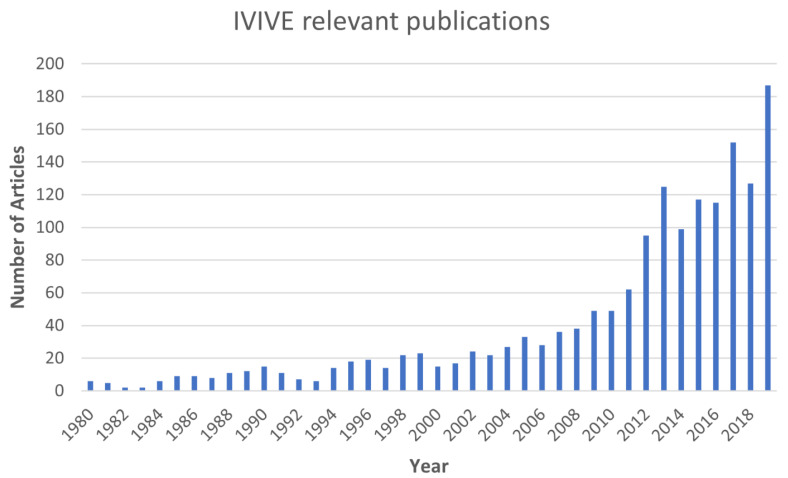
The number of articles found in the literature with the terms “*In vitro* to *in vivo* extrapolation” or “IVIVE”.

The literature search, as described in the Methods, returned 1138, 654, and 619 articles with PubMed, SCOPUS, and Web of Science, respectively. After combining the results from the 3 databases and removing the duplicates, 1680 articles remained. The number of IVIVE publications has significantly increased over the last decade (Figure 1).

The literature data set was further narrowed down by subject matter experts to 288 articles of direct relevance to this review via screening of titles and abstracts. Thereafter, these articles were grouped by the two main types of IVIVE applications: IVIVE of dosimetry and IVIVE of ADME parameters. An article that included both applications was counted in IVIVE of dosimetry, with the rationale that this application is more inclusive. Most articles fell into the category of IVIVE of ADME parameters (133 articles), rather than IVIVE of dosimetry (44 articles). The remaining articles fell into the other categories relevant to IVIVE, such as studies that compared *in vitro* and *in vivo* correlations.

### 4.2. IVIVE of Dosimetry

#### 4.2.1. Summary of Common Applications

**Figure 2:**
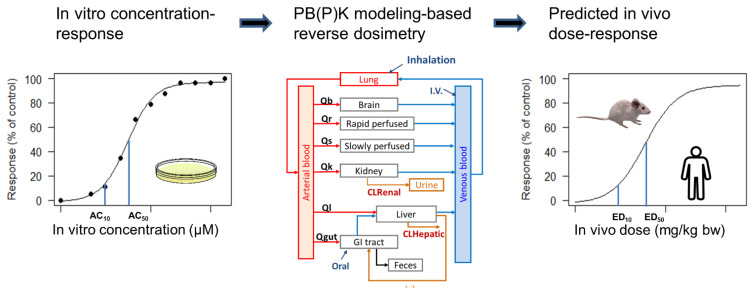
The process of IVIVE of dosimetry, figure adapted from Louisse et al. [108].

Figure 2 describes the process of converting an *in vitro* concentration associated with bioactivity to an external exposure level. Despite the general consensus that *in vitro* and *in silico* approaches hold great potential in revolutionizing toxicity testing and risk assessment [104], one of the key barriers to accepting the use of *in vitro* toxicity testing data to inform risk evaluations is the inability to relate the nominal assay concentration to a relevant *in vivo* exposure metric. The feasibility of employing a simplified PK model in an IVIVE approach to approximate the lowest effect levels for chemicals based on *in vitro* data was first tested in a pilot study on 35 chemicals [71], immediately followed by an effort that expanded the approach to 239 chemicals [31]. A PK model that incorporated hepatic clearance, renal (non-metabolic) clearance, and plasma protein binding was used to predict an external dose that would result in the respective steady-state plasma concentration, which corresponded to some *in vitro* bioactive concentration. This predicted dose has been referred to in a variety of ways as equivalent administered dose (EAD), administered equivalent dose (AED), or oral equivalent dose (OED) in the case of oral exposure [31,85,105]. For those chemicals with *in vivo* PK data, the approach was demonstrated to be reasonably predictive, with overestimation of plasma concentrations at a given external dose occurring for all but a few chemicals. In this case, such an overestimation was considered ultimately protective of human health. Depending on the assumptions used, *in vitro* bioactivity can be a conservative surrogate for *in vivo* toxicity, with relatively weak quantitative correlation between the two [70,72,73,105,106].

Meanwhile, Aylward and Hays [106] directly compared *in vitro* bioactive concentrations in the ToxCast database to *in vivo* plasma concentrations associated with the no-observed-adverse-effect level (NOAELs) and lowest-observed-adverse-effect levels (LOAELs) in traditional toxicological studies of laboratory animals or chronic human exposure reference values and plasma concentrations in human biomonitoring studies. Their assessment, which was limited to five chemicals, showed that *in vitro* bioactivity concentrations were similar to the range of *in vivo* concentrations associated with the transition from non-adverse to adverse responses [106]. Turley and colleagues conducted a case study of two indirect food additive chemicals, and showed that OEDs derived from ToxCast bioactivity data and IVIVE were similar to or lower than LOAELs and NOAELs in animal studies [66].

Beyond these high-throughput IVIVE (HT-IVIVE) applications, parallel efforts focusing on specific chemical spaces have been presented. Tonnelier and colleagues used IVIVE to assess bioaccumulative compounds covering pharmaceuticals, plant protection products, and industrial chemicals and demonstrated that metabolic clearance, plasma protein binding, and renal excretion are the main factors in determining whether bioaccumulation will occur [107]. Louisse and colleagues used a PBPK model for rat and human to convert *in vitro* concentration–response data of all-*trans*-retinoic acid into *in vivo* dose–response data [108], which were then used to derive a benchmark dose (BMD) [99,108,109]. Davidsen and colleagues estimated psychoactive substance exposures based on hair and whole blood biomarker concentrations, using both well-stirred and parallel tube models, to provide a basis for toxicokinetic understanding of ketamine analogues [110]. 

#### 4.2.2. Challenges and Additional Considerations for IVIVE of Dosimetry

One challenge associated with *in vitro* toxicity testing is distinguishing the disruption of specific biomolecular targets or pathways from generalized disruption of cellular machinery that can lead to cell stress and cytotoxicity. Multiple attempts have been made to characterize the bioactivity seen *in vitro*, which may be separated into a cytotoxic burst of non-specific activity and more focused effects on particular molecular targets [111,112,113]. Knowledge of the general cell stress response and cytotoxicity could help inform non-specific or common mechanisms, such as necrosis and regenerative proliferation; whereas knowledge of the specific molecular targets could help inform specific modes of action [112].

Zhang and colleagues suggested that IVIVE of toxic effects should be considered, in addition to IVIVE of dosimetry [46]. For toxic effects involving systemic regulation, organism-level PD models are needed to extrapolate *in vitro* toxicity pathway perturbation to *in vivo* adverse outcomes [114,115,116]. By linking PBPK and PD models, *in vitro* POD can then be extrapolated to external doses for expected exposure scenarios and relevant toxic endpoints [46,85,117].

IVIVE of dosimetry has also been used to improve correlation between *in vitro* bioactivity and *in vivo* toxicological endpoints such as *in vitro* lowest-observed-effect concentration (LOEC) versus *in vivo* lowest-observed-effect level (LOEL) [73]; however, the overall utility of IVIVE to assess toxicological risk depends on the characterization of experimental variability for *in vivo* and *in vitro* endpoints and the assessment of uncertainty and inter-individual variability in pharmacokinetic parameters [70,72,118,119,120].

Another challenge that can increase the uncertainty in IVIVE of dosimetry is the lack of a well-defined *in vitro* dose metric to describe the *in vitro* dose–response relationship. The traditional use of nominal concentration as a dose metric may not be appropriate as many factors can reduce the bioavailable and biological effect dose to levels far below the nominal concentration [36]. Non-specific migration to plastics and binding to media constituents (for example, serum proteins, lipids) have been documented, and test chemicals may evaporate, degrade, or be metabolized. All these factors can result in an underestimation of potency [121]. This information can be obtained by measuring chemical distribution in different tissue compartments or by mathematical model predictions. Several *in vitro* kinetic models have been proposed to convert nominal concentrations to free chemical concentrations in the well, largely based on physicochemical properties, such as log K_ow_ (n-octanol–water partition coefficient) [34,35,122,123,124]. Proença and colleagues have compared and assessed the performance of these mathematical models to predict free concentration [125]. Although these models show promise, a more thorough evaluation has been hindered by a lack of available experimental data.

**Figure 3:**
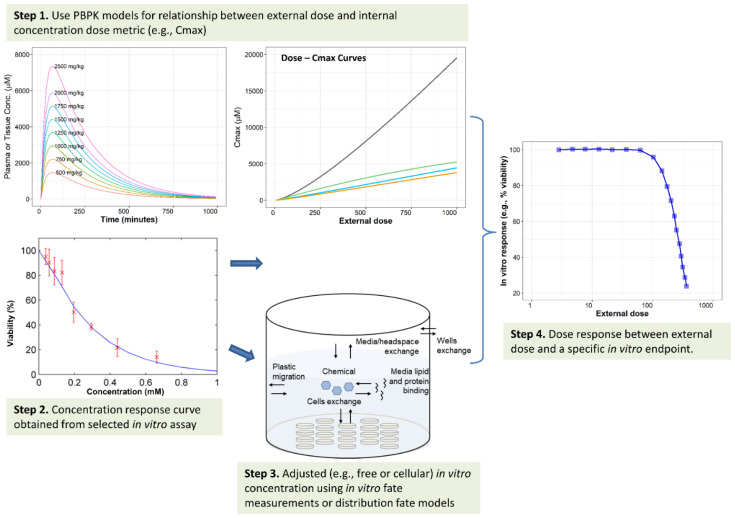
Consideration of *in vitro* kinetics in IVIVE of dosimetry. Step 1. Execute the PBPK model at the time point of interest at multiple doses to obtain chemical distribution in plasma and tissue compartment. Then, use the dose–response curve to determine the relationship between the external dose and Cmax or other internal dose metric (e.g., AUC) in plasma or selected tissue (e.g., liver). Step 2. Concentration–response curve obtained from selected *in vitro* assay. Nominal concentration is used for plotting. Step 3. Using appropriate *in vitro* kinetic models, adjust the *in vitro* nominal concentration in the testing well to free medium or cellular concentration. Step 4. Combine the external dose–Cmax curve form Step 1 and *in vitro* concentration–response curve (Step 2 or Step 3) to obtain a relationship between external dose and *in vitro* endpoint. Adapted from Paini, et al. [39].

A workflow for IVIVE of dosimetry that considers the *in vitro* kinetics is presented in Figure 3. This workflow is adapted from work by Louisse et al. [99], Punt et al. [126], and Caroline et al. [127]. Briefly, in Step 1 (Figure 3), a PBPK model can be used to predict relevant internal concentration dose metric, such as peak plasma or tissue concentration (Cmax) or time-integrated area under the plasma concentration vs. time curve (AUC), in plasma or a tissue over time and across a wide range of external doses. The dose metric may be selected based on a proposed mode of action or an *in vitro* endpoint of interest. For example, for relating to a cytotoxicity assay using hepatocytes, an external dose–peak liver concentration curve is predicted. In Step 2, *in vitro* toxicity testing can generate an *in vitro* dose–response curve. In parallel, in Step 3, the *in vitro* nominal concentration in the testing system can be adjusted and converted to a free medium or a cellular concentration with appropriate mathematical models or measurable analytics. Either nominal or adjusted concentrations may be used as effective *in vitro* concentration, which is assumed to be equivalent to the selected *in vivo* internal concentration dose metric (e.g., Cmax). Finally, in Step 4, the external dose–*in vitro* response curve is generated using the above assumption, from which a POD can be derived to inform risk assessment.

### 4.3. IVIVE of ADME Parameters

IVIVE has also been used to refer to scaling ADME properties measured in species- or population-specific *in vitro* systems to *in vivo* conditions, such as metabolic clearance, absorption, and bioavailability. IVIVE is a critical component of bottom-up PBPK models [76,88,128,129,130,131]. Compared to building a PBPK model primarily based on observed *in vivo* data (a top-down approach) [132,133], the bottom-up PBPK models are built mainly relying on *in vitro* and *in silico* data [88,134]. A large portion of the IVIVE literature discusses using IVIVE for predicting various ADME parameters. Drawing from earlier PBPK modeling and parameter estimation research efforts, a 2014 expert-driven workshop suggested that conservative default assumptions might be used in place of chemical-specific *in vivo* data for IVIVE [135]. While this is a reasonable approach, the advent of large *in vitro* datasets characterizing at least some key aspects for non-pharmaceutical compounds have opened up additional avenues for exploration and model refinement from the use of conservative default assumptions [31,75,100].

#### 4.3.1. Summary of Common Applications

The liver is the primary organ of metabolism and clearance from the body. A variety of *in vitro* platforms have been used to measure intrinsic metabolic activities of liver enzymes and are scaled to *in vivo* parameters for use in PBPKs. These platforms include recombinant enzymes [136], human liver microsomes [137], and primary hepatocytes [138]. Other experimental systems such as dynamic 3D bioreactors [139], coupled microfluidic systems, and vascularized human organ chips [140] have also been proposed as *in vitro* models to perform IVIVE of hepatic clearance. To scale *in vitro* intrinsic metabolic clearance, the data need to be normalized to units such as mg per protein per million cells, and then appropriate scaling factors to units such as L/hour need to be applied.

In addition, metabolic rate constants in non-hepatic tissues such as the gut, kidney, and lung can also be measured *in vitro* and extrapolated to *in vivo* [21,141,142,143,144]. Comparable to approaches for the liver, lung and kidney microsomes were used to estimate metabolism in non-hepatic tissues [144]. Kunze and colleagues showed that the transport measurement of a porcine proximal tubule cell line can be applied to predict human renal clearance as the net result of glomerular filtration, tubular secretion, and tubular reabsorption for multiple-class chemicals [21]. Wambaugh and colleagues suggested that the role of the extrahepatic metabolism may be more pronounced for non-pharmaceutical compounds [76].

*In vitro* cell-based systems are also commonly used to examine the passive and active transport mechanisms influencing permeability/absorption, distribution, and elimination of chemicals or their metabolites. These assay systems vary in complexity and can make use of membrane vesicles, cell lines transfected with relevant transporters, or other more sophisticated models. The specific *in vitro* parameters can also vary but will consider passive and active permeability and typically assume saturable Michaelis–Menten kinetics to help determine the rate of accumulation in the tissues or efflux from the tissues based on the direction of the transport. The model is the varied binding affinities of a substance with different transporters in the same system; however, utilizing *in vitro* systems is an efficient, cost-effective, and faster way of estimating the relevant parameters required to complement PBPK models. Among the most commonly used cells in assessing drug permeability and transport for predicting intestinal absorption are Caco-2 cells derived from a human colon adenocarcinoma and which express many active transporters [13,145]. The correlation between the *in vitro* apparent permeability coefficients across Caco-2 cell monolayers and the fraction of chemicals absorbed is well established [15]. Additionally, the *in vitro* cell-based systems based on the human embryonic kidney cell line 293 (HEK293) or Madin–Darby canine kidney (MDCK) cells have been commonly used to simulate the transport of substances in the kidney to examine the impact of renal transport mechanisms on their elimination [146].

For other parameters describing ADME properties such as drug penetration across the blood–brain barrier and unbound tissue to plasma partition coefficients, IVIVE approaches have also been developed using cell-based penetration models or *in vitro* membrane partitioning and were shown to be successfully extrapolated to *in vivo* settings [147,148]. Several review articles and the new OECD guidance on PBK modeling provide more details on *in vitro* and *in silico* approaches and tools, as well as read-across methods, for IVIVE of ADME parameters [13,15,101,149,150].

#### 4.3.2. Evaluations and Additional Considerations for IVIVE of ADME Parameters

The evaluation of PBPK models parameterized using IVIVE can be performed by comparing predictions from IVIVE-linked PBPK models with *in vivo* observations of internal dose metrics, such as steady-state concentration (Css), Cmax, or AUC [76,136]. Common evaluation metrics used for comparing model predictions to observations include average fold-error and absolute average fold-error [148,151], exposure overlap coefficients [152], percent error thresholds, and evaluating whether the observations fall within the 5th to 95th percentiles of model predictions [136]. Imperfect but statistically significant correlations have been observed, consistent with the idea that the available *in vitro* methods describe some key, but not all-encompassing, processes governing ADME. Wang found that, for six out of seven prototypical CYP3A inhibitors, the predicted Cmax fell within two-fold of the *in vivo* observations [128]. Wambaugh and colleagues also found that the predicted Css for 40% of ToxCast chemicals examined as of 2015 were within ~3.2-fold of the literature values [88].

*In vitro* extrapolated *in vivo* clearances are commonly found to underpredict *in vivo* hepatic clearances [14]. Disconnects among *in vivo* extrapolated clearances from distinct *in vitro* systems have also been observed and recently evaluated [153]. Thus, several strategies for scaling factors have been proposed in the literature; these strategies tend to be *in vitro* system-specific [154]. These scaling factors, such as intersystem extrapolation factors and relative activity factors, primarily aim to correct for any differences in enzyme activity between *in vitro* and *in vivo* systems [136,154]. Correction factors, commonly used for correcting drug binding in the incubations, plasma, and in the whole liver, are additional methods used for improving the predictive accuracy of the IVIVE-based PBPK modeling [14]. The underestimation of whole-body metabolism when using hepatic data may be more pronounced for non-pharmaceutical chemicals, potentially due to greater extrahepatic metabolism for chemicals not planned for use as therapeutics [76].

While most IVIVE applications have been developed for human health risk assessment, applications for non-human species are also available. Nichols and colleagues [81,82,83] have developed methods for measuring rainbow trout liver metabolism *in vitro* and incorporated metabolism information into PBPK models via appropriate scaling factors to derive more realistic estimates of fish bioconcentration factors. Stadnicka-Michalak and colleagues [155] correlated acute lethality of organic chemicals (log Kow between 0.5 and 7) in cultured fish gill cells with fathead minnow LC50s. Their approach used PK modeling of the cell culture system to derive time-dependent cellular concentrations of the target compound *in vitro* and compared these values to internal concentrations *in vivo* predicted with whole-body PBPK models. Wetmore et al. [72] found that human and rat fraction unbound in plasma and hepatic clearance were significantly but not perfectly correlated, while Black et al. [156] observed limited agreement among humans, rats, and trout.

### 4.4. Employing IVIVE to Predict In Vivo Toxicity

The IVIVE approach has also been applied for evaluating, at the screening level, the safety or toxic potential of environmental chemicals [6,31,100]. For example, Mebust and colleagues [157] combined a biophysical model of chromosomal damage, which was developed from *in vitro* data, with a dosimetric model to predict cancer incidences in rats exposed to radiation. Leonard and colleagues [42] used a PBPK-PD model to predict oral intake doses resulting in reduction of thyroid hormones by 10% for six drugs and environmental chemicals that inhibit thyroid peroxidase enzyme. With the advance of more sophisticated or more physiological *in vitro* and biomarker methods, more robust IVIVE predictions have been developed in recent years to support applications such as investigating cardiac safety of drugs [158] and studying the effects of metabolism on drug efficacy [159]. More omics-oriented PD predictions or enzyme-expression behaviors have been found in recent publications, providing potential substitutions for *in vivo* tests [6,160].

## 5. Case Examples from the Literature

This section highlights a few case examples of IVIVE applications from the literature. The case examples are considered more relevant to regulatory risk assessment based on the input from members of the IVIVE–WG.

### 5.1. For Prioritization

High-throughput pharmacokinetic models based on chemical properties and *in vitro* high-throughput data have been applied by the pharmaceutical industry in preparation for human clinical trials [128,134,136]. HT-IVIVE approaches have also been coupled to *in vitro* bioactivity data as an alternative to animal testing for evaluating the safety or toxic potential of environmental chemicals [31,100]. As described in the previous section, Wetmore and colleagues performed IVIVE on ToxCast bioactivity data to enable the comparison of ToxCast-based OEDs to human exposure or *in vivo* reference doses [31,72]. These comparisons demonstrated that the *in vitro* assays generally provided a conservative (that is, lower) POD estimate compared to those derived from *in vivo* studies, likely due to conservative assumptions made in the PK model (such as restrictive clearance) and lower threshold for bioactivity [31,70,73,105]. Based on the comparisons, chemicals with low margins of exposure are put on the priority list for further assessment. This HT-IVIVE approach, relying on hepatic clearance and plasma protein binding as critical determinants of internal dose estimations [31,100] has gained reasonable acceptance in the toxicology community as a prioritization tool in chemical risk assessment [70,161].

Subsequent efforts have been made to further explore the application of HT-IVIVE in chemical prioritization. As an important step toward estimating plausible biological interactions in a high-throughput risk assessment framework, Sipes and colleagues applied *in vitro* or *in silico* derived toxicokinetic parameters (for example, hepatic clearance and plasma protein binding) to perform IVIVE and exposure likelihood assessment to the entire Tox21 federal collaboration chemical screening data set, which provides a framework to relate *in vitro* toxicology data rapidly and quantitatively to chemical exposures [41]. To incorporate toxicodynamic variability in HT-IVIVE, Abdo and colleagues used human-population-based *in vitro* cytotoxicity screening data and comparative population genomics analyses to evaluate individual variability in responses to toxicants [162]. Wetmore and colleagues measured *in vitro* clearance rates for 13 cytochrome P450 and 5 uridine 5′-diphospho-glucuronysyltransferase isozymes using recombinantly expressed enzymes for selected ToxCast chemicals and incorporated the isozyme-specific clearance rates into an IVIVE model that captures known differences in isozyme expression across several life stages and ethnic populations. This approach allows for the estimation of subpopulation-specific OEDs that can be directly compared to subpopulation-specific exposure estimates [77]. Wambaugh and colleagues assessed toxicokinetic measurement uncertainty and variability in HT-IVIVE by developing a Bayesian method to provide chemical-specific uncertainty estimates for fraction unbound (fu) and intrinsic hepatic clearance (Clint) and also used Monte Carlo simulation to address both measurement uncertainty and biological variability into IVIVE [75]. A rough trend may be argued that toxicodynamic variability is greater than toxicokinetic variability, which is in turn greater than toxicokinetic uncertainty for *in vitro* TK methods; however, there are individual chemical cases or sets of measurements where this order may be inverted, for example, due to difficulties working with the chemicals *in vitro* [163].

Efforts are ongoing, but more research is needed for integration of AOP in IVIVE and IVIVE of mixtures. El-Masri applied IVIVE to estimate maternal exposures that would yield fetal blood levels equivalent to the chemical activity concentration of selected *in vitro* HTS assays related to the most sensitive AOP. A life-stage PBPK model was used to convert fetal blood levels to maternal exposures, which were then compared to potential exposure levels for deriving AOP-based margins of exposure [164]. Compared to individual chemical applications, HT-IVIVE for mixtures remains largely unexplored. Abdo and colleagues applied IVIVE to convert *in vitro* cytotoxic concentrations to OEDs for two pesticide mixtures with similar ranges of *in vitro* cytotoxicity and identified nominal differences in the margins of safety, suggesting the necessity of including IVIVE and potential human exposures in risk evaluation [162]. Valdiviezo and colleagues investigated the concentration-dependent effects of chemical interactions on toxicokinetic parameters using 20 pesticides (both individually and as equimolar mixtures) and observed that IVIVE using mixture-derived toxicokinetic data produced more conservative estimates of activity-to-exposure ratios as compared to using data from single chemical experiments [165]. 

### 5.2. Developmental Toxicity

Several *in vitro* assays have been developed as alternatives for developmental toxicity testing of chemicals in animal models [166,167]. These include cellular assays (such as the rat limb bud micromass test), the embryonic stem cell test, and other culture assays (such as whole embryos of rat, frog, chicken, and zebrafish). In these assays, *in vitro* readouts including toxicogenomic and metabolomic data have been identified as biomarkers for potential use in predicting developmental toxicity *in vivo* [167,168]. However, *in vitro* findings alone, commonly used for hazard identification, have not always been reflective of *in vivo* toxicity to support quantitative chemical risk assessment [169,170]. Louisse and colleagues [167] used embryotoxicity as an example to discuss how *in vitro* effect data could be translated to *in vivo* conditions with different approaches: (1) classify whether a compound has a weak or strong embryotoxic potential using statistical models; (2) predict relative embryotoxic potencies for a group of structurally related compounds using QSAR models and read-across approaches; and (3) convert *in vitro* effect concentrations to equivalent administered *in vivo* doses using PBPK modeling and IVIVE [167].

PBPK-IVIVE approaches are increasingly being applied to risk assessment, such as predicting developmental *in vivo* dose–response for the development toxicity of tebuconazole, an agricultural fungicide [171]. In this study, an adult rat PBPK model primarily parameterized by *in silico* and *in vitro* approaches was developed, and the model’s predictive performance was evaluated using available *in vivo* kinetic data. The authors had previously demonstrated that tebuconazole does not cross into the placenta [172]; it was therefore assumed that maternal blood concentration was an adequate surrogate for concentrations in fetal tissues. Maternal blood concentration was used as the target concentration equivalent to *in vitro* effect concentrations to perform IVIVE to predict the *in vivo* dose–response relationship [171]. BMD modeling was then applied to the extrapolated *in vivo* dose–response to estimate the lower bound values of the 95% confidence interval of the BMD associated with a 10% extra risk of adverse effect (BMDL10). The estimated BMDL10 value was less than the reported *in vivo* POD value by three-fold, demonstrating the potential to use this approach for conducting risk assessment without performing *in vivo* studies. This approach has also been used to predict developmental toxicity potential for other classes of developmental toxicants, such as glycol ethers in rats and humans [99], all-*trans*-retinoic acid in rats and humans [109], and phenols in rats [169].

PBPK-IVIVE approaches can also include population analysis to study the influence of inter-individual variabilities in developmental toxicity, as in the study of phenols conducted by Strikwold and colleagues [173]. In addition, dynamic life-stage PBPK models can be used to conduct IVIVE, as in the study that predicted embryotoxicity from ethanol exposure in various species, including humans, during critical windows of developmental toxicity [68].

### 5.3. Endocrine Effects

Many environmental chemicals have the potential to interact with hormone receptors and cause a variety of adverse health effects, posing regulatory challenges. To address these challenges, a growing body of international *in vitro* test guidelines have been established to address mechanisms and modes of action of endocrine-disrupting chemicals to assist in the safety assessment of this class of substances. Standardized methods to incorporate metabolic and PK aspects into these *in vitro* tests are necessary and still under development [174,175].

*In vitro* estrogen receptor (ER) assays targeted to different key events in the ER activation pathway, such as ER binding and dimerization, have been developed as alternatives for measuring estrogenic activity [176]. Several studies on IVIVE of dosimetry have been conducted to predict the lowest effect levels (LELs) in rodent uterotrophic assays using bioactive concentrations from *in vitro*-measured endpoints related to the ER signaling pathway. In one of the early studies [177], a population-based PK model was used to conduct IVIVE for two ER reference chemicals, estradiol and bisphenol A, and demonstrated that the OEDs estimated from the *in vitro* POD of an ER transactivation assay were lower than the LELs in rat uterotrophic assays. The result suggested that this ER transactivation assay may provide a more conservative hazard estimate for use in risk assessment. More recently, *in vitro* concentration–response data for the same chemicals obtained from various *in vitro* assays were translated into *in vivo* dose–response data using a PBPK model developed based on *in vitro* and *in silico* derived parameter values [178]. BMD analysis was subsequently performed on the predicted dose–response data to produce BMDL10 values, which were compared to those values derived from rodent uterotrophic assay data. One of the *in vitro* assays, the yeast estrogen screen assay, was identified as having the best potential to predict dose-dependent uterus growth induced by estrogenic chemicals [178].

Casey and colleagues [105] evaluated the performance of three PK models of different structure and complexity in IVIVE of dosimetry for a group of 29 ER agonists. They found little difference in model performance based on complexity, and demonstrated that simple adjustments, applied to account for *in vitro* intracellular exposure or chemical bioavailability, resulted in significant improvements in the predictive performance of all PK models tested [105]. In a later study, a human uterine cell estrogen response assay was used to estimate *in vivo* equivalent doses for a set of chemicals and found 19 out of 23 chemicals to have an EAD lower or equivalent to PODs in the rodent uterotrophic assay. This equivalency also suggests that an *in vitro* assay could provide a more conservative estimate for human health risk than the rodent uterotrophic assay [179]. Punt and colleagues applied an IVIVE approach to prioritize different polycarbonate monomers for their endocrine potencies by combining *in vitro* bioassay data with PBPK modeling. This study revealed a shift of relative potency between *in vitro*-measured potencies and IVIVE-based estimates, which is likely due to the influence of intestinal metabolism on the *in vivo* availability [180].

Compared to ER, there are fewer reports describing IVIVE analyses predicting androgen receptor activity. Kleinstreuer and colleagues conducted IVIVE of dosimetry using *in vitro* activity concentration predictions from an androgen receptor pathway model developed based on 11 high-throughput *in vitro* assays. It was demonstrated that IVIVE can be helpful in explaining the discrepancy in potency ranking seen between *in vitro* AR pathway model prediction and the *in vivo* Hershberger assay. Considering the variability seen in *in vivo* assays, it is suggested that the *in vitro* AR pathway model may better predict specific AR interaction and could rapidly and cost-effectively screen thousands of chemicals without using animals [67].

### 5.4. Case Examples of IVIVE of ADME Parameters

PBPK models can be built primarily based on observed *in vivo* data (a top-down approach) [132,133] or mainly rely on *in vitro* and *in silico* data (a bottom-up approach) [88,134]. Traditionally, development of PBPK models required significant resources, particularly experiments characterizing chemical concentration in tissues as a function of time, dose, and route of exposure [181]. As the traditional approach is not capable of keeping pace with the new toxicity testing paradigm, more bottom-up PBPK models are being developed based on a combination of results from *in vitro* and *in silico* methods [101].

PBPK models are intended to be fit-for-purpose, that is, explicitly simulating only the key biological and ADME processes and tissue compartments of interest while “lumping” less relevant aspects together. Considering this, it is important to define the objective of the model and identify the existing data gaps before searching for relevant *in vitro* data in literature or designing experiments to generate *in vitro* data for parameterizing PBPK models. Examples of specific applications of PBPK model parameterization and development using IVIVE of ADME parameters are described below.

Malmborg and colleagues provided an example citing the importance of conducting IVIVE for the various metabolically active tissues, including liver, gut, and blood, and integrating them using PBPK modeling, especially for drugs administered as a prodrug [182]. Wambaugh and colleagues relied on *in vitro* metabolism data to characterize active metabolism and developed a machine-learning model trained to predict the discrepancy between *in vitro*-based predictions and *in vivo* observations of PK [88]. Campbell and colleagues developed a hybrid computational fluid dynamic–PBPK model for naphthalene, in which the metabolic rates were determined experimentally using *in vitro* methods. The model supported cross-species dosimetry comparisons of naphthalene concentrations and tissue normalized rates of metabolism in the nasal respiratory and olfactory epithelium, lungs, and liver. This model predicted human equivalent inhalation concentrations corresponding to several NOAELs or LOAELs for noncancer effects of naphthalene observed in rats [141].

An example of IVIVE for predicting renal transport is the rat PBPK model of perfluorooctanoic acid (PFOA), in which published data generated using *in vitro* cell systems were utilized to establish the role of renal reabsorption in the elimination of PFOA via the involvement of several transporters [183]. The renal reabsorption prediction from the PBPK model was scaled and/or normalized from *in vitro* data, such as the Vmax (capacity) and Km (affinity) of basolateral and apical transporters measured in *in vitro* assays [184,185]. This approach also included separate relative activity factors of apical and basolateral transporters for males and females, which enabled the prediction of sex-based differences in renal transport and the elimination of PFOA. The PBPK-model-based predictions of the concentrations of PFOA in the liver, blood, and urine correlated with experimental data for both the male and female rats, indicating that *in vitro-*derived physiological descriptions of transporter-mediated renal reabsorption can reasonably predict sex-dependent elimination of PFOA.

Recently, new approaches have been developed for calculating *in vitro* unbound tissue to plasma partition coefficients using *in vitro* membrane partitioning, and the parameters were shown to be extrapolated *in vivo* to predict whole-body drug distribution using PBPK modeling [148]. In addition, a life-stage-specific PBPK model can be tailored using *in vitro* data for chemicals with sparse data sets for predicting dosimetry in different life stages, such as infants, children, pregnant and lactating women, and fetuses [68,186,187,188,189,190]. For example, *in vitro* methods can be used to determine differences in metabolic parameters among populations, ethnic groups, and ages, such as those based on differences in expression of cytochromes P450 and associated genetic polymorphisms, or adjusted from enzyme ontogeny or polymorphism [17,136,191,192,193,194].

### 5.5. IVIVE Application to Engineered Nanomaterials (ENMs)

One topic that has generated widespread research interest is IVIVE of studies using ENMs, including how to assess ENM dosimetry [195]. Similar approaches would typically also work for microplastic and nanoplastic particles with some exceptions (for example, larger microplastic particles becoming trapped during passage through an exposure system) [196]. For exposure systems that use deposition onto cells located at the air–liquid interface after aerosolization of dry powders or ENM suspensions, it may be possible to directly quantify the deposited concentration [197,198,199]. However, one complicating factor is that some ENMs, such as carbonaceous particles, may be more challenging to quantify at low concentrations in biological matrices [200,201]. In addition, it may be challenging to differentiate between particles that have simply deposited onto the cells but are not yet internalized since the amount removed may vary based on the washing procedure, which may potentially remove viable cells [197]. The intracellular concentration may be more directly comparable to the tissue concentration in *in vivo* studies than nominal concentration [197]. When cells are exposed by addition of an ENM suspension to the underlying basolateral media, it may also be possible to directly quantify the concentration associated with the cells.

For studies that use exposure in a submerged system where an ENM suspension is added to the overlying media, several computational models are available to estimate the concentration expected to reach the cells. For ENMs that do not dissolve, the *in vitro* sedimentation, diffusion, and dosimetry (ISDD) model has been widely used to estimate the ENM concentration delivered to the cells [202,203,204]. It should be noted that this concentration estimation may vary based on the method used to characterize the size distribution of the suspended particles, especially if there is significant agglomeration [205]. For ENMs where dissolution may occur, such as for silver ENMs, an improved ISDD model which also incorporates dissolution has been developed [206]. Overall, these models require measuring several parameters such as the particle size or size distribution and the effective density.

These measured or modeled concentrations during *in vitro* experiments can then be compared to modeled concentrations of the internal dose during the *in vivo* experiments such as by using the multi-path particle dosimetry model [207,208]. It is also possible to use lung burden measurements for IVIVE comparisons when historical data are available [209]. Performing lung burden measurements is an option in OECD standard methods (for example, TG 413 [210]) for particles that may be retained such as ENMs.

## 6. IVIVE Resources and Tools

### 6.1. Information Obtained from Literature and Agency’s Responses

The ICCVAM IVIVE-WG provided input for ICCVAM member agencies regarding the modeling tools or software each agency plans to use or has used for facilitating IVIVE analysis and decision making. The responses are summarized in the Table 3.

Additional data resources and tools for conducting IVIVE collected from the literature and the world wide web are summarized below.

### 6.2. Resources for Chemical Properties and In Vitro Data

#### 6.2.1. Resources for Chemical Properties Data

When experimental measurements are not available, physicochemical properties are needed for predicting tissue:plasma partition coefficients and membrane permeability [213]. There are several useful open resources for obtaining chemical properties. OCHEM (Online chemical database with modeling environment), https://ochem.eu/ (accessed on 20 October 2021), is a web-based system with a chemical information database and QSAR modeling framework [214]. OCHEM also includes toxicological alerts, a user guide, and tutorials. Other public sources for chemical information include ChemSpider [215] and ChEMBL [216]. EPA CompTox Chemicals Dashboard (https://comptox.epa.gov/dashboard, accessed on 20 October 2021) [217] also provides chemical-specific information, including chemical properties, *in vitro* bioactivity, toxicokinetic, and IVIVE predictions for more than 880,000 chemicals. Compared to other databases, the CompTox Chemicals Dashboard focuses on curated chemical structures, as designated by its underlying database, DSSTox [218]. DSSTox assigns a unique structure identifier (DTXSID) to each structure [219]. The curation of structures for DSSTox is intended to protect against inaccurate chemical identification, often observed in public repositories [220].

PubChem (https://pubchem.ncbi.nlm.nih.gov/, accessed on 20 October 2021) is a public chemical information resource at the U.S. National Library of Medicine (NLM), National Institute for Biotechnology Information (NCBI). It provides freely accessible chemical information that includes chemical name, molecular formula, structure, chemical and physical properties, biological activities, and safety and toxicity information for over 100 million unique chemical structures extracted from chemical substance descriptions contributed by data depositors [221,222]. NLM NCBI’s Bookshelf (https://www.ncbi.nlm.nih.gov/books/, accessed on 20 October 2021), also known as Books, provides no-cost online access to books and other documents, including those related to IVIVE, from U.S. Government agencies and other organizations around the world. [TOXNET (the TOXicology data NETwork) was retired in December 2019 as part of the reorganization associated with the NLM Strategic Plan (https://www.nlm.nih.gov/pubs/plan/lrp17/NLM_StrategicReport2017_2027.html, accessed on 20 October 2021). Most of TOXNET’s databases have been incorporated into other NLM resources such as PubChem and the NCBI’s Bookshelf or continue to be available elsewhere].

In a recent publication, Madden and colleagues summarized available resources relevant to the development of PBPK models [223]. This review distinguishes freely available versus commercial resources, and those that provide predicted versus measured values. Summarized in this review are resources for predicting external exposure, physicochemical properties, ADME properties, physiological or anatomical parameters, model structures for specific organs, PBPK modeling software, and similar chemical determination. Pawar and colleagues [224] compiled a systematic review and grouping of databases that can assist in chemical or drug safety assessment. This review provides a comprehensive list of the key *in silico* data resources relevant to chemical identity and properties, drug action, toxicology, exposure, omics, pathways, ADME properties, clinical data, and databases relating to animal alternatives in support of 3Rs policies. Also included is a list of previous review articles for identification of databases relevant to chemistry and toxicology [224].

#### 6.2.2. Resources for *In Vitro* ADME Data (Reviews or Multiple Topics)

From the articles returned by literature search, the IVIVE-WG also prepared Table 4 summarizing various *in vitro* ADME data that can potentially be used for IVIVE.

### 6.3. Models and Tools for PBPK Modeling and IVIVE

#### 6.3.1. Resources Explicitly Designed to Support IVIVE of Dosimetry and Related Activities 

The Integrated Chemical Environment (ICE: https://ice.ntp.niehs.nih.gov/, accessed on 24 March 2022), hosted by the National Toxicology Program Interagency Center for the Evaluation of Alternative Toxicological Methods (NICEATM), provides access to high-quality curated data and computational tools, including an IVIVE workflow, to facilitate the use of *in vitro* alternatives in chemical hazard identification and modeling [243]. Users are able to specify their own lists of chemical identifiers, choose from established reference sets, or combine the two approaches. These chemical inputs can then be used in searches for available data from legacy *in vivo* studies, mechanistically annotated *in vitro* data, *in silico* prediction models, and curated ToxCast or Tox21 data that incorporate analytical chemistry QC information and data processing flags to ensure high-quality curve fits and hit calls [244].

ICE-curated HTS data are available as an input into the IVIVE workflow, which relies upon a combination of models from the EPA’s httk R package and in-house PK simulations to translate *in vitro* activity concentrations into estimated EAD [105]. Multiple dosing routes (oral, intravenous, and inhalation), species parameterization (rat, human), and model structures (one-compartment, three-compartment, multi-compartment) with customizable exposure intervals and simulation lengths are available. Experimental data for plasma protein binding and intrinsic clearance are incorporated for about 1000 chemicals, while *in silico* predictions for these parameters are provided for the entire DSSTox Database of ~800k chemicals from the Open Structure-activity/property Relationship App (OPERA, https://github.com/NIEHS/OPERA, accessed on 24 March 2022) [245]. In addition to the curated HTS data, the ICE user has the option to upload their own *in vitro* bioactivity data for running the IVIVE workflow and obtaining results in graphical and tabular form. The predicted EADs can be compared to doses from animal studies by overlaying data points on the graphs, and the mechanistic target annotations are intended to facilitate biologically meaningful comparisons by helping the user select *in vitro* and *in vivo* assays that query similar pathways.

Other open-source web-based PBPK modeling tools include a web-based toolbox that contains generic PBK models for rats and humans developed by RIKILT Wageningen University and Research Center. This toolbox provides calculation tools to predict plasma protein binding and tissue:plasma distribution, which can be used for IVIVE of dosimetry [246].

As mentioned above, the CompTox Chemicals Dashboard currently provides *in vitro* bioactivity data for thousands of chemicals from ToxCast [48,247], Tox21 [47], and PubChem [221,222], as well as structure-based model predictions for tens of thousands more chemicals. To facilitate IVIVE, *in vitro* measure hepatic clearance and fraction unbound in plasma are reported for more than a thousand chemicals. In addition, the predicted volume of distribution, days to steady-state, PK half-life, and Css are provided under the IVIVE Table. The Css values are calculated assuming a 1 mg/kg/day rate and the 95th percentile of a distribution representing a population of healthy adults. These data are available for the chemicals listed at (https://comptox.epa.gov/dashboard/chemical_lists/HTTKHUMAN, accessed on 24 March 2022).

#### 6.3.2. Other Models and Tools for PBPK Modeling and IVIVE

Other than the web tools that are explicitly designed to support PBPK modeling and IVIVE, various commercial and open-source software, such as Simcyp/SIVA [248], Gastroplus [249], PK-Sim [250], httk [87], and Cloe PK [251], allow for performing IVIVE of ADME parameters and implementing it in PBPK modeling, and facilitating IVIVE of dosimetry. A thorough list of PBPK/IVIVE software and tools is summarized in the Supplemental Excel Sheet. Tools for IVIVE have different levels of complexity. More detailed predictions can be made for individual chemicals when more data are incorporated into a model, whereas general predictions are made for large groups of data-poor chemicals (as in high-throughput bioactivity screening) [24,100]. The higher-throughput approaches typically trade off certainty for speed and flexibility [69,70].

As discussed earlier, due to chemical partitioning to various components of the assay systems (for example, plastic, media proteins or lipids, head space, cells), the nominal concentration in the test medium does not always provide an adequate estimate of chemical potency when using *in vitro* activity to inform *in vivo* toxicity. To calculate the mass distribution of a chemical within the *in vitro* test system at equilibrium, Armitage et al. [122] published a mass-balance model that considers critical components of *in vitro* assay systems (such as % serum in media, media volume, cell number) along with the physicochemical properties of the test article. The Armitage model is implemented in Excel using the Visual Basic for Applications programming language, and has recently been implemented in the open-source R package “httk” [73]. To estimate the biologically effective concentration, Fischer and colleagues developed an equilibrium partitioning model to predict freely dissolved, cellular, and membrane concentrations in the Tox21 GeneBLAzer bioassays for a set of organic chemicals [35]. The model can readily be applied to diverse *in vitro* bioassays as an Excel workbook that provides all relevant system parameters and a generic bioassay setup.

Attempts to evaluate generic PK modeling approaches find the best case for predictions is closer to a factor of 3 [88,128,129], which is larger than the average error factor of 2 discussed by the International Programme on Chemical Safety (IPCS) of the World Health Organization [252]. In addition, generic PK modeling approaches generally perform worse for predicting the time course of plasma or tissue concentrations than for summary statistics such as peak or time-integrated concentration [76,86,130]. To better facilitate the evaluation of generic PK models, Sayre and colleagues [120] developed a public database of published chemical concentration vs. time data along with standardized formats for reporting the outcome of PK experiments.

Moreover, a population-specific IVIVE-based PBPK model can be a valuable tool for analyzing human biomonitoring studies in support of human health risk assessment (Sharma et al., 2018). IVIVE calculations reported on the CompTox Chemicals Dashboard are performed using the open-source R package “httk” (https://cran.r-project.org/web/packages/httk/index.html, accessed on 24 March 2022) and include a Monte Carlo simulation for population variability to identify the adults who obtain the upper 95th percentile highest plasma concentrations from the same 1 mg/kg bw/day exposure [87]. “httk” is itself a resource for IVIVE, as it includes generic models and chemical-specific data for simulation and statistical analysis of chemical toxicokinetics. Chemical-specific data are curated from the peer-reviewed scientific literature for both humans and other species relevant to toxicology such as rats (>200 compounds) [73]. “httk” uses methods for predicting tissue:plasma partition coefficients (PCs) and volume of distribution that have been calibrated to better reflect *in vivo* observations [87]. The Monte Carlo sampler for human variability is based upon biometrics described by the U.S. Centers for Disease Control and Prevention National Health and Nutrition Examination Survey [74]. The Monte Carlo sampler also allows for propagating chemical-specific parameter uncertainty [75]. In addition to the CompTox Chemicals Dashboard, graphical interfaces to the predictions from “httk” are also provided by ICE [243] and the Population Life-course exposure to health effects model (PLETHEM) [253].

## 7. Agency Needs, Areas of Research Needed, and Future Opportunities

### 7.1. Agency Needs, Gaps, and Uncertainty in IVIVE

A workshop report by Bell and colleagues [29] identified aspects of regulatory decision making where IVIVE may already be appropriate, such as screening data-poor chemicals for potential toxicity, improving dose selection, developing data-derived uncertainty factors, and supporting the development of testing strategies. The report also identified areas of need for IVIVE applications, such as guidance on model complexity, the development of evaluation criteria, examination of differences between pharmaceutical and non-pharmaceutical compounds, databases for *in vivo* and *in vitro* toxicokinetic data, and harmonization among scientific institutions.

The IVIVE–WG gathered information, from ICCVAM member agencies, on specific needs for implementing IVIVE approaches in regulatory risk assessment, data gaps or uncertainty that prevents using IVIVE in risk assessment. Table 5 summarizes this information.

The historic application of IVIVE and reverse dosimetry focuses on chemical prioritization using *in vitro* points of departure from various assays [31]. To go beyond prioritization and screening decisions, most regulators require consistent approaches and good understanding of the advantages, disadvantages, and uncertainties of different approaches. Some agencies also need approaches that demonstrate effectiveness for mixture risk assessment and more sophisticated approaches that provide target tissue estimates.

Concerns on gaps or uncertainty in IVIVE approaches from agencies include understanding of mechanistic relevance of NAMs to *in vivo* outcomes, robust consideration of chemical domain of applicability, inter-individual variability, and uncertainty in parameter estimation. Challenges faced by multiple agencies also include identifying artifacts in an *in vitro* system, identifying toxic moiety in an *in vitro* system, and selection of internal *in vivo* concentration corresponding to the *in vitro* concentration. For example, shall *in vitro* half-maximal activity concentration (AC_50_) be regarded to be equal to maximum or steady-state plasma concentration? *In vitro* assays that lack full metabolic competence limit evaluations on the effects of parent compounds. In parallel, current high-throughput IVIVE approaches also only focus on predicting the toxicokinetics of parent compounds.

Long-term efforts to address these limitations will require additional research conducted in parallel with more historical efforts already underway, such as developing a database of PK models, metabolic relationships between chemicals, and *in vitro* distribution information. Conduct of such research is already underway at several U.S. and international agencies and is detailed below.

### 7.2. Efforts to Address Needs and Future Opportunity

IVIVE research typically occurs in one of three broad areas: chemical-specific, physiologic, and population or life-stage. The area requiring the largest investment is the first, as chemical-specific toxicokinetic and *in vitro* distribution information is a base requirement that can then be applied across all endpoints of interest. Physiologic inputs are finite by comparison, once described. For example, for all applicable species, tissues, they can be retained in an established database [87]. Population or life-stage based libraries track ontogenetics and variability for populations of interest that can then be used to inform the physiologic inputs employed in modeling and simulation efforts [74]. As outputs are generated, evaluation that makes use of available *in vivo* data can inform the assessment of uncertainty and variability [75]. It follows that research to inform these areas proceeds on four fronts: data generation, informatics, modeling, and evaluation.

Data generation has and will continue to collect chemical-specific *in vitro* plasma protein binding and hepatic clearance data to expand the tested chemical space on the CompTox Chemicals Dashboard [31,100]. Furthermore, the implementation and evaluation of additional assays that capture extrahepatic clearance, passive permeability, transport kinetics, and chemical affinity for specific metabolizing enzymes are an important next step to not only improve predictions of *in vivo* toxicokinetics but to inform target tissue distribution more effectively. For *in vitro* distribution, the number of chemicals characterized is relatively small [122]. Large systematic efforts are needed to collect *in vitro* kinetic data, such that correlation with physicochemical properties may be evaluated and better understood. Finally, in some cases, limited *in vivo* data are still being generated to establish the suitability of IVIVE for chemical classes, where existing *in vivo* data are scarce.

Informatics for IVIVE includes developing ontologies for describing key data types and then organizing and curating existing data into machine-readable formats such that algorithmic analysis for patterns is possible. Efforts are already underway for organizing *in vivo* pharmacokinetic concentration time-course data [120] and human variability in metabolic enzyme expression [254]. Additional efforts might include the following: mapping parent–metabolite relationships, annotating data from *in vitro* pharmacokinetic assays, organizing data identifying the enzymes that interact with chemicals, continuing to develop and expand libraries of pharmacokinetic models [150], and structuring datasets characterizing the partitioning of chemicals to materials encountered *in vitro*. The international harmonization of databases and development of open-source tools will also be important to establish consistent approaches for IVIVE.

As *in vitro* assays proliferate and new chemical classes are investigated, models must similarly expand. Existing PBPK models for IVIVE have focused on organic compounds and mostly the parent compound. However, new efforts are examining systematic approaches to metals and PK models that generally allow for chemical transformation, including cycling [255]. In addition, for PK models, the rapid growth in informatics has allowed the development of many approaches relating chemical structure features to important properties, including *in vitro* PK measurements [41,78,84]. Quantitative structure–property relationships (QSPRs) are rapidly developing, and both new models and consensus predictors based on multiple models should be expected [79,256]. *In vitro* distribution models need to be expanded to consider chemical ionization, time-dependent distribution, and repeated dosing.

Ultimately, all of these models may only be used with confidence when their predictive accuracy has been statistically evaluated. Many risk assessment paradigms make use of default uncertainty factors to take into account extrapolation across species and differences in route of exposure [257,258,259]. To make use of IVIVE in decision-making [29,135], rigorous statistical evaluation of IVIVE or *in vitro*–*in vivo* correlation are needed to quantitatively determine the confidence with which they may be applied [24,33,70,73,76,260].

For most regulators, “validation” is a legal determination of acceptability for decisions that impact public health, the environment, and commerce [261,262]. To support validation, scientists need to determine when and how to apply a methodology and the level of confidence for a given class of chemicals for the intended purposes. Regulatory agencies may also have qualification programs for NAMs related to specific contexts of use, to drive a comprehensive collaborative avenue of evaluation towards acceptance. For example, the Model-Informed Drug Development Meeting Pilot Program initiated in a pilot version at FDA aims to provide a path for direct specialized interactions between drug developers and the agency on suggested NAMs to address product-specific applications [263]. Other qualification programs for potential drug development tools are available at the FDA to address gaps or inefficiencies in drug development that relate to regulatory needs [264]. As NAMs gain acceptance in regulatory fields, guidance may be developed for industry if needed to facilitate their implementation and acceptance. Information to support these efforts may include specialized contributions, data, and methodologies from relevant academic, private, and public stakeholders [265].

Gaining further insight and understanding of uncertainties through continued research and testing should remain a priority to build transparency and confidence. The development of harmonized databases will increase data accessibility and use by domestic and international entities. At the same time, the development of open-source methods and software packages will motivate increased consistency of application and the predictive accuracy of *in silico* and *in vitro* methods for IVIVE.

## Figures and Tables

**Table 1:** Specific risk assessment applications that can involve the use of IVIVE.

Agency/Organization	Use of *In Vitro to In Vivo Extrapolation* (IVIVE) in Risk Characterization	Use of IVIVE or *In Vitro* Data Outside of Quantitative Risk Characterization
Agency for Toxic Substances and Disease Registry (ATSDR)	Application of IVIVE approaches would require the ability to derive health guidance values using high-throughput *in vitro* data. Several uncertainties and assumptions remain; hence, IVIVE is not used in health assessments.	*In vitro* data are used or potentially used as weight of evidence.
U.S. Food and Drug Administration Center for Food Safety and Applied Nutrition (FDA/CFSAN)	Use IVIVE to develop physiologically based pharmacokinetic (PBPK) models, specifically to account for metabolism in the liver and transport in the kidney.	Not applicable (N/A)
FDA Center for Drug Evaluation and Research (FDA/CDER)	The role of IVIVE in risk assessment has generally been limited to relating *in vitro* human ether-à-go-go-related gene (hERG) channel assay results to the risk of QT prolongation and PBPK modeling. Following established decision trees in dedicated guidance [59], *in vitro* data can be used to predict drug–drug interactions and therefore dismiss the need for clinical trials. It is anticipated that appropriately constructed IVIVE algorithms will play a critical role in assessing the utility of new approach methodologies (NAMs) proposed to be used in risk assessment, which may include the support of starting dose selection in first-in-human trials of products using the Minimum Anticipated Biological Effect Level [60].	*In vitro* data can predict efficacy of drugs and estimate doses to use with high potential in the field of rare diseases [61].
Consumer Product Safety Commission (CPSC)	Has not used the approach but could use the information during any applicable risk evaluation; the approach could be used in a weight of evidence approach for risk assessments.	N/A
U.S. Environmental Protection Agency, Office of Pesticide Programs (EPA/OPP)	Use IVIVE to perform a rapid risk screening for chemicals without *in vivo* toxicity data [62] or to support a weight of evidence approach to identify data needs or to derive extrapolation factors [63].	Identify chemicals that act on a common mechanism.
U.S. Department of Defense (DoD)	Various applications use IVIVE to derive human-relevant numbers to address operational human toxicity issues providing informed assessment of risk. This approach has also been used in a corroborative weight of evidence evaluation of hazard (comparisons across various data streams).	N/A
National Institute of Environmental Health Sciences, National Toxicology Program (NIEHS/NTP)	N/A	Perform hazard characterization. Use IVIVE to estimate external doses needed to achieve blood levels that equate to the identified *in vitro* potencies. The approach is applied to multiple species including human.
European Union Reference Laboratory for Alternatives to Animal Testing (EURL ECVAM)	N/A—does not conduct regulatory risk assessments.	Development of case studies to explore and illustrate applicability of *in vitro* data and IVIVE.

**Table 2:** Summary of current Agency’s publications or guidance documents that are related to IVIVE.

Agency/Organization	Publications or Guidance Documents
ATSDR	ATSDR does not have guidance on IVIVE.
CPSC	CPSC has no guidance document related to IVIVE. There is a proposed Guidance on Alternative Test Methods and Integrated Testing Approaches, 86 FR 16704, 31 March 2021.
DoD	The DoD has no specific guidance on IVIVE implementation; however, other guidance frameworks are currently being developed.
EPA	Guidance Documents: [49,64,65] Publications grouped into the following categories: Workshop report, review or perspective related to IVIVE: [29,66]; IVIVE application for specific biological pathway: [67,68]; IVIVE application using HTS assays: [25,31,41,69,70,71,72]; Evaluation of uncertainly and variability of IVIVE approach: [73,74,75,76,77]; PK parameter prediction and evaluation: [78,79,80,81,82,83,84]; Open-source tools for PBPK modeling and IVIVE: [85,86,87,88]; General statements of chemical risk assessment goals including IVIVE: [7,89,90,91,92].
NIEHS/NTP	Publications: [29,41,73,76]
FDA/CDER	Publications: [57,93,94] Guidance Documents: In Vitro Drug Interaction Studies—Cytochrome P450 Enzyme- and Transporter-Mediated Drug Interactions Guidance for Industry [59]; Clinical Drug Interaction Studies—Cytochrome P450 Enzyme- and Transporter-Mediated Drug Interactions Guidance for Industry [95]; Physiologically Based Pharmacokinetic Analyses—Format and Content Guidance for Industry [96]; Guidance for Industry Pulmonary Tuberculosis: Developing Drugs for Treatment [97]; The importance of a rigorous IVIVE algorithm to the qualification of a NAM for embryofetal developmental toxicity is captured in Annex 2 of ICH S5(R3) Detection of Reproductive and Developmental Toxicity for Human Pharmaceuticals: Guidance for Industry [98].
European Commission/EURL ECVAM	There is no specific guidance on IVIVE so far, but various approaches have been reviewed or explored [39,43,99,100]. OECD PBK model guidance describes IVIVE approach illustrated with several case studies [101]; EURL ECVAM workshop highlighted the need to develop guidance on constructing PBK models without the use of *in vivo* data to support IVIVE applications [54]; OECD “Guidance Document on Good In Vitro Method Practices (GIVIMP)” [50] guidance also reports use of IVIVE approach; European chemicals agency (ECHA) publishes reports emphasizing the important role of (Q)IVIVE in *in vitro*-based hazard identification and providing recommendations for (Q)IVIVE implementation [102]; The Scientific Committee on Consumer Safety (SCCS) adopted one guidance document on the safety assessment of nanomaterials in cosmetics, in which IVIVE is required for safety assessment mostly or entirely based on *in vitro* test results [103].
Health Canada	Science approach document on bioactivity exposure ratio: application in priority setting and risk assessment [58].

**Table 3:** The models or software tools agencies and organizations plan to use or make available to facilitate IVIVE analysis in decision making.

Agency/Organization	Models or Software Tools
ATSDR	Models or software tools such as PBPK modeling have been used for dosimetric adjustments in the minimal risk level (MRL) determination process.
CPSC	There are no current plans to use models or software for facilitating IVIVE analysis and decision-making.
DoD	Current software use runs the spectrum of options. Current legacy software is used for PBPK (e.g., acslX for PBPK modeling); widely available software (e.g., R, also for PBPK modeling); high-throughput toxicokinetics (httk) R package; molecular docking and deep learning (TensorFlow); AOP wiki; STRING, REACTOME, OECD QSAR Toolbox, and BIOVIA software packages; and tools developed within image analysis tools for cell cultures.
EPA/ORD	Developed httk R package [87]; Simcyp^TM^ for PBPK modeling; PBPK model knowledgebase [150]; Database of PK time-series data and parameters [120].
NIEHS/NTP	No decision-making. Use httk R package, GastroPlus & ADMET Predictor (Simulations Plus), as well as the Integrated Chemical Environment (ICE) tool.
European Commission/EURL ECVAM	No decision-making. Use httk R package (for the Accelerating the Pace of Chemical Risk Assessment [APCRA] project); Berkeley Madonna PBK model; explored application of the Wetmore IVIVE approach [31] and the BMD approach in a reverse dosimetry way; ongoing work from EFSA on the toxicokinetic plate and EPAA project on IVIVE. An IVIVE EPAA project [211,212] conducted by Health and Safety Executive, UK, is ongoing, which is based on work from McNally et al. [43] (led by G. Loizou). It will provide a tool to translate *in vitro* concentration–response relationships to *in vivo* dose–responses, determine *in vivo* benchmark dose (BMD) values from the translated data, and compare the predicted *in vivo* BMD to existing experimental BMD values used in chemical safety assessments by a regulatory agency.

**Table 4:** List of resources for *in vitro* assay data.

*In Vitro* Assay Data Type	Data Summary	References
Overview or summary of *in vitro* and *in silico* data	Comparison of metabolic clearance assay systems; discussion of computational systems with built-in *in vitro* biochemical scaling	[29]
	*In vitro* ADME methods overview	[135]
	Kidney enzymes, transporters, scaling factors	[143,225,226]
	This review has an emphasis on test systems and dosimetry in the respiratory tract.	[227]
	As part of an assessment of QSAR quality and reproducibility, 80 models of 31 ADME-related endpoints were identified.	[228]
	A summary table in the Supplementary Materials of Patel et al. [228] notes published sources for *in vitro* data and QSARs pertaining to oral absorption (7 data sets), distribution across the blood–brain barrier (1 data set), and metabolism data (8 data sets: *in vitro* metabolic clearance, Vmax, and Km).	[228]
	Summaries of resources of ADME data sets, models, and predictive software (designated as freely available or commercial products); while these tables do not emphasize *in vitro* data, these resources are well represented.	[223]
	Review of “high-throughput toxicokinetics”—the combination of *in vitro* chemical-specific methods with generic toxicokinetic models for IVIVE	[85]
*In vitro* data: metabolism in hepatocytes, microsomes, and purified enzymes	Hepatocyte, microsomal, and purified (non-recombinant) hepatic enzyme data assembled by Pirovano et al. for QSAR development	[229,230]
	Literature curated intrinsic clearance data from pooled hepatocyte suspensions for 1015 chemicals measured using human hepatocytes and 225 chemicals using rat hepatocytes. Included in R package “httk”	[87]
*In vitro* scaling data for scaling liver metabolism	Age-specific data (5-year bins, for adult humans aged 20–95 years old) for microsomal protein content of liver and liver weight used in Simcyp	[231]
	“Age-dependent protein abundance of cytosolic alcohol and aldehyde dehydrogenases in human liver.” (neonates to adults)	[232]
	Human hepatic microsomal protein yields and hepatocellularity collated from multiple sources. Weakly statistically significant inverse relationship to age; no relationship with gender, smoking, or alcohol consumption	[233]
	Human hepatic CYP content (total, and per isoform, for 7 isoforms; *n* = 60 subjects); rat and human hepatocyte numbers and microsomal protein yield	[234]
	Human hepatic CYP content central tendencies and variation (total and per isoform, 10 isoforms, 42–350 white subjects); reviews of data on impact of disease, age, sex, environment, and genetics on hepatic clearance	[235]
	Distribution of hepatic microsomal protein yields for 128 adult (Chinese) humans	[236]
	Human hepatic microsomal protein yields (20 adults from the United Kingdom)	[237]
	Hepatic metabolism scaling factors for rainbow trout (microsomal protein yield, hepatocellularity, liver S9 yield, and CYP content (CYP2M1, CYP2K1, and CYP3A27)	[83]
	Population variability in hepatocellularity, liver blood flow, liver volume and liver density for estimating *in vivo* hepatic clearance from *in vitro* data. Implemented in R package “httk”	[74]
Partition coefficients (PCs)	A decision tree was described to choose the best predicted tissue partition coefficients for a certain physicochemical space, selecting among 6 algorithms, based on a 122-drug training set.	[238]
	Reports Quantitative Property Relationship (QPPR) models for human and rat blood:air PCs for diverse volatile organic chemicals	[239]
	Examines and compares the relative accuracy, strengths, and limitations of 7 published models for human tissue–air and 10 models for tissue–blood PCs. The most accurate models for each category were identified.	[240]
	Reports a QSAR model for predicting physicochemical and biochemical properties of industrial chemicals of various groups	[241]
	Evaluation of QSAR predictions for 964 experimentally derived chemical–tissue PC combinations (143 chemicals, 12 tissues) with calibration and uncertainty quantification; Data and results are implemented in R package “httk”.	[242]

**Table 5:** Agency Needs and Concerns on Gaps or Uncertainty in IVIVE approaches.

Agency/Organization	Agency Needs	Concerns on Gaps or Uncertainty
ATSDR	Harmonized methods for risk assessors. Success stories to help strategic training and thinking. An electronic version of methodology. Understanding advantages and disadvantages or uncertainties of different approaches. Agency does not develop regulatory risk assessments.	Gaps in the understanding of toxicity mechanisms involved. Agreement and differences in interpretation of data for same endpoint using multiple assays. Uncertainties and assumptions in the transformation of *in vitro* dose. Derivation of health guidance values using *in vitro* assay results.
FDA/CFSAN	To establish a consistent approach for IVIVE.	Consistent and consensual criteria for evaluating IVIVE approaches for specific purposes. Lack of experiment data for PK model validation. Refinement of a validated IVIVE approach for fit-for-purpose application.
FDA/CDER	IVIVE needed to support the qualification of NAM(s) associated with specific regulatory context(s) of use.	Concerns will depend on the context of use being addressed by a NAM being qualified and include: Data quality; Availability of clinical data; Understanding the mechanistic relevance of the NAM regarding the *in vivo* or clinical setting being modeled.
CPSC	The method needs to be effective for mixture risk assessment.	Demonstration of effectiveness for mixture risk assessment.
EPA/OPP	Determining the needs for additional in vivo studies. Providing additional data for a weight of evidence approach to estimate data-derived extrapolation factors [63].	Challenges in linking *in vitro* concentration to relevant *in vivo* dose metric. Challenges in identifying toxic moiety in an *in vivo* system unless the *in vitro* system has metabolism capability.
EPA Office of Pollution Prevention and Toxics (OPPT)	Determine plausible route(s) of exposure: dermal, inhalation, oral.	Many chemicals are considered rapidly with only structure and physicochemical properties available. No time for even *in vitro* measurements of TK. Must rely on QSAR.
EPA/ORD	Rapidly estimate doses based on the bioactivity data that EPA generated. Best practices for use case, for example, when to use which in silico models for predicting input parameters for IVIVE.	Current high-throughput pharmacokinetic methods need to be expanded to better characterize tissue distribution, particularly for active transport barriers such as blood–brain barrier, placenta, and lactation. Development of statistics-ready databases of information from the peer-reviewed literature, including pharmacokinetic models, tissue concentration vs. time data, metabolic relationships between chemicals, in vitro toxicokinetic measurements, and in vitro distribution information.
DoD	Currently accepts IVIVE data, verified or validated NAMs. Methods that predict interorgan relationships or effects.	Applicable endpoints—acute lethality is significant for connecting to historical databases and for narrow uses with specific chemical classes (e.g., chemical agent). Biomarkers of effect, e.g., carboxyhemoglobin levels, behavioral or cognitive deficits (sleep deprivation or chemical intoxications), stress, are valuable endpoints, although notably difficult to predict. Organ specific endpoints such as pulmonary edema, ischemia (cardiac or brain), neurotransmitter alterations. Ability to test for interorgan effects (e.g., neuroendocrine, neurodevelopmental).
NIEHS/NTP	Agency does not develop regulatory risk assessments.	The standard issues with IVIVE might be explored further, e.g., domain of applicability, parameter estimation, uncertainty, inter-individual variability, accuracy, sensitivity, and specificity.
European Commission/EURL ECVAM	Agency does not develop regulatory risk assessments.	Artifacts in *in vitro* systems. Uncertainty factors needs to be established to extrapolate.

## Data Availability

Not applicable.

## Supplementary Materials

The following supporting information can be downloaded at: https://www.mdpi.com/article/10.3390/toxics10050232/s1, Table S1: Concise list of general and IVIVE approach terms commonly used in scientific and regulatory arenas; Supplemental Excel Sheet: List of software and tools for PBPK modeling and IVIVE.

## Disclaimer

This article may be the work product of an employee or group of employees of ATSDR, CPSC, DoD, DOE, DOI, EPA, FDA, NIOSH, NIST, USDA, or other organizations; however, the statements, opinions, or conclusions contained therein do not necessarily represent the statements, opinions, or conclusions of ATSDR, CPSC, DoD, DOE, DOI, EPA, FDA, NIOSH, NIST, USDA, the United States government, or other organizations. Inotiv-RTP staff provide technical support for NICEATM but do not represent NIEHS, NTP, or the official positions of any federal agency. Dr. Sweeney is a contractor working with the Air Force Research Laboratory/711th Human Performance Wing, and the views expressed in this paper are her own and do not necessarily reflect the views of the Air Force, the Department of Defense, or her employer, UES, Inc. Hakkinen retired from NIH NLM in 2020 and is now an NIH Special Volunteer in Toxicology and Environmental Health Sciences at NCBI. For help with Bookshelf, PubChem, and other NCBI databases, contact the Help Desk (https://support.nlm.nih.gov/support/create-case/ (accessed on 24 March 2022).

## Abbreviations

3Rs	to replace, reduce, and refine (or replacement, reduction, and refinement of) the use of animal models
ADME	absorption, distribution, metabolism and excretion
ATSDR	Agency for Toxic Substances and Disease Registry
AUC	area under curve
BMD	benchmark dose, the dose of a chemical that is required to achieve a predetermined response of a toxicological effect
BMD10	derived benchmark dose that is associated with a 10% extra risk of adverse effect in the exposed test animals
BMDL10	the lower bound of 95% confidence interval on BMD10
CFSAN	FDA Center for Food Safety and Applied Nutrition
Cmax	the highest concentration of a chemical in the blood or a tissue after a dose is given
CPSC	U.S. Consumer Product Safety Commission
Css	steady-state concentration
DoD	U.S. Department of Defense
EAD	equivalent administered dose
ENM	engineered nanomaterial
EPA	U.S. Environmental Protection Agency
ER	estrogen receptor
EURL-ECVAM	European Union Reference Laboratory for Alternatives to Animal Testing
FDA	U.S. Food and Drug Administration
HTS	high throughput screening
HT-IVIVE	high throughput in vitro to in vivo extrapolation
ICCVAM	Interagency Coordinating Committee on the Validation of Alternative Methods
ICE	Integrated Chemical Environment
IVIVE	in vitro to in vivo extrapolation
IVIVE-WG	ICCVAM in vitro to in vivo Extrapolation Workgroup
LOAEL	low observed adverse effects level
log Kow	the n-octanol / water partition ratio or coefficient
NAM	new approach methodology
NIEHS	National Institute of Environmental Health Sciences
NIST	National Institute of Standards and Technology
NLM	U.S. National Library of Medicine
NOAEL	no observed adverse effect level
NOEL	no observed effect level
NTP	National Toxicology Program
OECD	Organisation for Economic Co-operation and Development
OED	oral equivalent dose
OPP	EPA Office of Pesticide Programs
QSAR	quantitative structure activity relationship
PD	pharmacodynamics
PK	pharmacokinetics
PBK	physiologically based kinetics
PBPK	physiologically based pharmacokinetics
PBTK	physiologically based toxicokinetics
PFOA	perfluorooctanoic acid
POD	point of departure
ToxCast^TM^	Toxicity forecaster
Tox21	Toxicology in the 21st century
