# Supplementary material for: IVIVE: Facilitating the Use of In Vitro Toxicity Data in Risk Assessment and Decision Making
Source: Toxics. 2022 May 1;10(5):232. doi: 10.3390/toxics10050232 (PMC9143724; doi:10.3390/toxics10050232)
Supplement: Supplementary file 1 [file toxics-10-00232-s001.zip › IVIVEms_SuppTableS1_controlled vocabulary_Final.pdf]

Multiple definitions of IVIVE in literature

- IVIVE of dosimetry – converting an in vitro dose (usually a point of departure related to in vitro activities) to an in vivo external dose or exposure, and kinetic models are usually involved to estimate an external dose
- IVIVE of ADME parameters – converting an in vitro-measured kinetic parameter (e.g., dermal absorption rate, hepatic metabolism rate) to the corresponding in vivo kinetic parameter, often these parameters are used in kinetic models (to upscale)

Summarize commonly used terms that are involved with IVIVE approach

There are two main steam definitions representing the IVIVE approach. To make the distinctions clear it is proposed IVIVE of dosimetry and IVIVE of ADME parameters (IVIVE category). Based on these definitions we have established a list of key controlled vocabulary. This was done in order to align and harmonize the terminologies used in different sectorial area and communities (e.g. regulators vs scientist) that are using the IVIVE approach. Table 1 reports a summary of these key terminologies and their respective "affiliation" to an IVIVE category (General, IVIVE of dosimetry and/or IVIVE of ADME parameters).

Supplemental Table S1. Concise list of general and IVIVE approach terms commonly used in scientific and regulatory arenas.

Consolidated Term + Acronym	Consolidated Definition	IVIVE Category "Affiliation"
Alternative test methods	Test methods developed to reduce, refine, or replace current animal test methods. Modern approaches to toxicity testing are preferred over animal testing and include: In vitro methods, which are performed outside living organisms; In silico methods, which are performed using computers and computer simulation; Chip models, which include human cell cultures placed on computer chips for study.	General definition
Benchmark approach	The BMD approach is applicable to all toxicological effects. It makes use of all of the dose–response data to estimate the shape of the overall dose–response relationship for a particular endpoint. The BMD is a dose level, estimated from the fitted dose–response curve, associated with a specified change in response, the benchmark response (BMR).	General definition
Bespoke toxicokinetic model	A toxicokinetic model developed for a specific chemical. These models may be empirical or physiologically based. The models are often optimized with respect to structure (for example, including physiological processes relevant to chemical disposition or use) as well as with respect to parameters. Bespoke models often reflect both the chemical and the evaluation data used.	General definition
Biokinetics	The study of the way in which particular substances move through the body and are metabolized.	General definition

Concentration (C)	The amount of a chemical or substance present in a particular quantity of soil, water, air, food, blood, hair, urine, breath, or any other media. Examples of units µM, mg/m^3, mg/L, ppm, percentage (%).	General definition
Domain of applicability (DA)	A well-defined region of chemical properties within which a method is expected to be chemical-appropriate and outside of which there is either no expectation or the expectation of failure.	General definition
Dosage (of a substance)	Total amount of a substance administered to, taken up, or absorbed by an organism, organ, or tissue. Dose divided by product of mass of organism and time of dose. Units in mg or mg/kg/day, may be used as a synonym for dose. However, other parameters are needed to characterize the exposure to xenobiotics. The most important are the number of doses, frequency, and total time period of the treatment.	General definition
Equivalent administered dose (EAD)	Dose produces the in vivo plasma or tissue concentration equivalent to an in vitro effective concentration. Usually reported in mg/kg-day.	General definition
Evaluation	The use of rigorous statistical methodology to compare the predictions of a model with the observed data. Evaluation may be characterized by a number of summary statistics such as root mean squared error, fraction of variance explained, or absolute fold error.	General definition
Forward dosimetry	Predicting biomarker concentrations at a given exposure concentration.	General definition
Generic toxicokinetic modeling	Chemical-independent models of key absorption, distribution, metabolism, and excretion processes in a manner that can be parameterized for many chemicals. These models may be empirical or physiologically based. Generic models allow statistical evaluation using with extant in vivo data such that the statistical performance may be extrapolated to other chemicals.	General definition
httk (high throughput toxicokinetics)	The combination of generic toxicokinetic models and in vitro toxicokinetic data such that chemical-specific predictions of toxicokinetics may be rapidly made.	General definition
Human toxicokinetic adjustment factor (HKTF)	Chemical-specific adjustment factors (also called CSAF) that are used to model interindividual variability in toxicokinetics in population-based IVIVE models. Unitless	General definition
In silico	Data generated, performed and analysed using computational methods by means of computer simulations.	General definition
In vitro	Any experiment that is not conducted in a living, multi-cellular organism. Is performed outside living organisms. May include "ex vivo" experiments such as tissue explants, isolated cells such as hepatocytes, etc.	General definition
In vitro single dose	In vitro testing in which test chemical is only tested at a single concentration, often this is a predetermined limit (i.e., 100 µM) concentration, or it may be driven by the chemical's solubility. Usually reported in µM, ppm.	General definition
In vitro toxicity testing	Toxicity testing that is not conducted in a living, multi-cellular organism. May include "ex vivo" experiments such as tissue explants, isolated cells such as hepatocytes, etc.	General definition
In vitro toxicokinetics	Generation of chemical-specific data that informs toxicokinetics using in vitro methods. It enables converting an in vitro dose to an in vivo internal dose (e.g., free concentration in plasma)	General definition
In vivo	Within a living organism or body.	General definition
In vivo dose-response	An in vivo testing system in which multiple doses are tested.	General definition
In vivo single-dose	In vivo testing in which test chemical is only tested at a single dose often this is a predetermined limit (i.e., 100 µM) concentration, or it may be driven by the chemical's solubility. Also known as a "limit test". It can be reported in mg/kg, mg/mL, ppm.	General definition
Integrated Approaches to Testing and Assessment (IATA)	Science-based approaches to characterize chemical hazard that use a systematic analysis of existing data coupled with in vitro and in silico approaches to fill data gaps.	General definition

Intracellular concentration	Concentration that reaches the inside of the cell. Usually reported in µM, ppm, weight/weight, wet weight.	General definition
Limit Test	In vivo testing in which test chemical is only tested at a single dose often driven by a regulatory limit or the chemical's solubility. Usually reported in mg/kg, mg/mL, ppm.	General definition
mathematical model	An explicit description of physical, chemical, and/or biological phenomena that allows the organization of existing data and make predictions that can be evaluated. All models should be evaluated and have a defined domain of applicability.	General definition
Mode of Action (MoA)	A sequence of events, identified by research, which explains an observed effect, including biokinetic and toxicodynamic effects.	General definition
New approach methodologies (NAMs)	In vitro and/or in silico methods, workflows, or adverse outcome pathways developed to reduce, refine, or replace animal tests.	General definition
Nominal concentration	The quantity of chemical added divided by the volume of the exposure medium. Potential unit in µM.	General definition
Pharmacodynamics (PD)	The study of the biochemical and physiologic effects of a drug on biological systems.	General definition
Pharmacokinetics (PK)	The study of the time course of drug absorption, distribution, metabolism, and excretion. Quantitation of the time course of chemical absorption, distribution, metabolism, and elimination.	General definition
Physiologically Based Biokinetic models (PBBK) Physiologically Based Kinetic models (PBK) Physiologically Based Toxicokinetic models (PBTK)	Synonyms of PBPK; Is a mathematical description of the organism physiology and the uptake, absorption, distribution, metabolism and excretion (ADME) of drugs and/or chemicals in the different organs of the body interlinked by blood flow (human or other animal species). Units depends on the output.	General definition
Physiologically Based Pharmacokinetic (PBPK) Models	A model that incorporates pharmacokinetic and mechanistic data into the extrapolation process. This model requires extensive data and is becoming commonly used. Units depends on the output.	General definition
Point of Departure (PoD)	The concentration where the response exceeds the assay-dependent noise threshold. Usually in µM.	General definition
Quantitative structure–activity relationship (QSAR) and structure–property relationship (QSPR) models	Mathematical regression or classification models used in the chemical and biological sciences and engineering, to predict the physicochemical, biological and environmental fate properties of compounds from the knowledge of their chemical structure. Unit depends on the outputs.	General definition
Reverse dosimetry (or Reverse toxicokinetics)	Reverse dosimetry uses a pharmacokinetic model along with measured biomarker concentrations to determine the plausible exposure concentrations.	General definition
Toxicodynamics (TD)	Describes the dynamic interactions of a toxicant with a biological target and its biological effects	General definition
Toxicokinetics (TK)	The study of the processes by which potentially toxic substances are handled in the body. This involves an understanding of the absorption, distribution, metabolism and excretion of such substances. Toxicokinetics is essentially the study of "how a substance gets into the body and what happens to it in the body." Before this term was used, the study of the kinetics (movement) of chemicals was originally conducted with pharmaceuticals and the term pharmacokinetics became commonly used. Similarly, toxicology studies were initially conducted with drugs. Toxicokinetics deals with what the body does with a drug when given a relatively high dose relative to the therapeutic dose.	General definition

Uncertainty	The distribution of values of a parameter that are plausible given the available data and the assumed model. Uncertainty reflects a lack of knowledge that may be reduced with additional data. If additional data do not reduce the uncertainty this calls into question both the data and the assumed model.	General definition
Uncertainty Factors (UF)	Factors used in the calculation of acceptable human or environmental exposures, which are applied to data from laboratory experiments or epidemiology studies. Factors of 10 are normally used to account for uncertainties in the data on which risk assessments are made. Similar to safety factors.	General definition
Variability	Biological, chemical, or physical variation in the value of a parameter that reflects the real world distribution of that parameter. Variability cannot be reduced with more data, only better characterized.	General definition
Weight of evidence (WoE)	A process in which all of the evidence relating to a decision is evaluated based on its strength and quality.	General definition
Activity Concentration (AC_X)	Chemical concentration that produces X% of the maximum activity in the assay. Where X% of the maximal activity concentration. (e.g. AC50 - half-maximal activity concentration). Usually reported in µM.	IVIVE of dosimetry
Adverse Outcome Pathways (AOPs)	An analytical construct that describes a chain of biological events at different levels of organization from molecular up, that leads to a chemical-independent adverse outcome on the organismal or population level.	IVIVE of dosimetry
Benchmark concentration (BMC)	Statistically calculated lower 95% confidence limit on the concentration that produces a defined response (called the benchmark response or BMR, usually 5 % or 10 %) for an adverse effect compared to background, often defined as 0 % or 5%. Usually reported in µM or ppm.	IVIVE of dosimetry
Benchmark dose (BMD)	The minimum dose of a substance that produces a clear, low level health risk, usually in the range of a 1-10% change in a specific toxic effect such as cancer induction. Usually reported in mg/kg or mg/mL or ppm.	IVIVE of dosimetry
Effective Concentration (EC50)	The EC50 is the dose at which 50% of the maximum effect is produced or the concentration of drug at which the drug is half-maximally effective. Usually reported in µM.	IVIVE of dosimetry
Free concentration	The concentration of unbound test chemical in the medium.	IVIVE of dosimetry
Inhibition concentration (IC50)	Concentration of inhibitor where the response is reduced by half. Usually reported in µM, mM.	IVIVE of dosimetry
In vitro dose-response	The relationship between the amount of a chemical administered and the subsequent effect in an in vitro testing system in which multiple test concentrations are tested.	IVIVE of dosimetry
LED (lowest effective dose)	Lowest dose of a chemical inducing a specified effect in a specified fraction of exposed individuals. Usually reported in mg/kg, mg/mL, ppm.	IVIVE of dosimetry
LEL (lowest effect level, also LOAEL and LOEL)	In a series of dose levels tested, it is the lowest level at which an effect (adverse) is observed in the species tested. Usually reported in mg/kg, mg/mL, ppm.	IVIVE of dosimetry
LOAEL (lowest observed adverse effect level)	Lowest dose at which there was an observed toxic or adverse effect.	IVIVE of dosimetry
LOAEL (lowest observed adverse effect level)	The lowest dose or exposure level of a chemical in a study at which there is a statistically or biologically significant increase in the frequency or severity of an adverse effect in the exposed population as compared with an appropriate, unexposed control group. Usually reported in mg/kg, mg/mL, ppm.	IVIVE of dosimetry

Lowest-observed-effect level (LOEL)	Lowest concentration or amount of a substance (dose), found by experiment or observation, that causes any alteration in morphology, functional capacity, growth, development, or life span of target organisms distinguishable from normal (control) organisms of the same species and strain under the same defined conditions of exposure. Usually reported in mg/kg, mg/mL, ppm.	IVIVE of dosimetry
NOAEL (no observed adverse effect level)	An exposure level at which there are no statistically or biologically significant increases in the frequency or severity of adverse effects between the exposed population and its appropriate control; some effects may be produced at this level, but they are not considered as adverse or precursors to adverse effects. In an experiment with several NOAELs, the regulatory focus is primarily on the highest one, leading to the common usage of the term NOAEL as the highest exposure without adverse effect. Usually unit in mg/kg, mg/mL, ppm.	IVIVE of dosimetry
Cytosolic protein per gram liver (CPPGL)	A scaling factor used for extrapolating data from cytosolic proteins per gram of liver. Usually reported in mg/g.	IVIVE of ADME parameters
Hepatocytes per gram per liver (HPGL)	A scaling factor used for extrapolating data from hepatocytes per gram of liver. Usually reported in cells/g.	IVIVE of ADME parameters
In vitro hepatocyte clearance	In vitro Intrinsic clearance rate using a mixed donor pool of primary hepatocytes. Usually reported in µl/min/10^6 cells.	IVIVE of ADME parameters
In vitro intrinsic clearance	Intrinsic clearance rate of an in vitro system, usually using hepatic microsomes or hepatocytes. Usually reported in µL/min-mg of microsomal protein or µl/min/10^6 cells.	IVIVE of ADME parameters
In vitro metabolic clearance	Metabolic clearance rate of an in vitro system, usually using hepatic microsomes or hepatocytes. Usually reported in µL/min-mg of microsomal protein or µl/min/10^6 cells.	IVIVE of ADME parameters
In vitro microsomal clearance	In vitro metabolic clearance rate using microsomal protein. Usually reported in µL/min-mg of microsomal protein.	IVIVE of ADME parameters
Ki	Inhibitory constant. Usually reported in µmol, mmol, etc.	IVIVE of ADME parameters
Km	The substrate concentration at half of maximum velocity i.e. Vmax (Michaelis Menten enzyme kinetics). Usually reported in µmol, mmol, etc.	IVIVE of ADME parameters
Microsomal protein per gram of kidney (MPPGK)	A scaling factor used for extrapolating data from microsomes per gram of kidney. Usually in mg/g.	IVIVE of ADME parameters
Microsomal protein per gram per liver (MPPGL)	A scaling factor used for extrapolating data from hepatic microsomes per gram of liver. Usually in mg/g.	IVIVE of ADME parameters
Vmax ($Vmax_{in\ vitro}$ or $Vmax_{in\ vivo}$)	Maximum rate of metabolism in an in vitro system or an in vivo system. Usually represented in µmol/min; mmol/min, etc.	IVIVE of ADME parameters
Blood to plasma ratio	The ratio of chemical concentration in the whole blood to the chemical concentration in the plasma. This is a function of chemical binding to red blood cells and the hematocrit. This parameter (Rb:p) is important when relating blood flows to plasma concentrations. Unitless ratio.	IVIVE of dosimetry & IVIVE of ADME parameters
Concentration at Steady State (Css)	Steady-state blood concentration. Usually reported in µM.	IVIVE of dosimetry & IVIVE of ADME parameters
Flow (Q)	Blood flow. Usually in L/h.	IVIVE of dosimetry & IVIVE of ADME parameters
Fraction of Chemical Unbound (fub)	Fraction of chemical unbound in the blood. Unitless.	IVIVE of dosimetry & IVIVE of ADME parameters
Fraction of Chemical Unbound (fup)	Fraction of chemical unbound in the plasma. Unitless.	IVIVE of dosimetry & IVIVE of ADME parameters

In vivo intrinsic clearance	Intrinsic clearance is the ability of the liver to remove drug in the absence of flow limitations and binding to cells or proteins in the blood. Usually reported in L/h or L/h/kg-BW.	IVIVE of dosimetry & IVIVE of ADME parameters
In vivo intrinsic clearance	The ability of the liver to remove drug in the absence of flow limitations and binding to cells or proteins in the blood. Usually reported in L/h or L/h/kg-BW.	IVIVE of dosimetry & IVIVE of ADME parameters
Volume of distribution (Vd)	Volume of distribution (V_d) is a primary PK parameter that relates drug concentration measured in plasma or blood to the amount of drug in the body and is used to characterize drug distribution. The apparent volume of distribution (VD) is the total volume of body fluids in which a toxicant is distributed. The VD is expressed in litres (L).	IVIVE of dosimetry & IVIVE of ADME parameters
